# Genome-wide identification of *Thellungiella salsuginea* microRNAs with putative roles in the salt stress response

**DOI:** 10.1186/1471-2229-13-180

**Published:** 2013-11-15

**Authors:** Quan Zhang, Chuanzhi Zhao, Ming Li, Wei Sun, Yan Liu, Han Xia, Mingnan Sun, Aiqin Li, Changsheng Li, Shuzhen Zhao, Lei Hou, Jean-François Picimbon, Xingjun Wang, Yanxiu Zhao

**Affiliations:** 1 College of Life Sciences, Shandong Normal University, Jinan 250014, PR China; 2 Bio-Tech Research Center, Shandong Academy of Agricultural Sciences; Shandong Provincial Key Laboratory of Crop Genetic Improvement, Ecology and Physiology, Jinan 250100, PR China

**Keywords:** *Thellungiella salsuginea*, Salt stress, miRNA identification, Solexa sequencing, Expression analysis

## Abstract

**Background:**

MicroRNAs are key regulators of plant growth and development with important roles in environmental adaptation. The microRNAs from the halophyte species *Thellungiella salsuginea* (salt cress), which exhibits extreme salt stress tolerance, remain to be investigated. The sequenced genome of *T. salsuginea* and the availability of high-throughput sequencing technology enabled us to discover the conserved and novel miRNAs in this plant species. It is interesting to identify the microRNAs from *T. salsuginea* genome wide and study their roles in salt stress response.

**Results:**

In this study, two *T. salsuginea* small RNA libraries were constructed and sequenced using Solexa technology. We identified 109 miRNAs that had previously been reported in other plant species. A total of 137 novel miRNA candidates were identified, among which the miR* sequence of 26 miRNAs was detected. In addition, 143 and 425 target mRNAs were predicted for the previously identified and *Thellungiella-*specific miRNAs, respectively. A quarter of these putative targets encode transcription factors. Furthermore, numerous signaling factor encoding genes, defense-related genes, and transporter encoding genes were amongst the identified targets, some of which were shown to be important for salt tolerance. Cleavage sites of seven target genes were validated by 5’ RACE, and some of the miRNAs were confirmed by qRT-PCR analysis. The expression levels of 26 known miRNAs in the roots and leaves of plants subjected to NaCl treatment were determined by Affymetrix microarray analysis. The expression of most tested miRNA families was up- or down-regulated upon NaCl treatment. Differential response patterns between the leaves and roots were observed for these miRNAs.

**Conclusions:**

Our results indicated that diverse set of miRNAs of *T. salsuginea* were responsive to salt stress and could play an important role in the salt stress response*.*

## Background

*Thellungiella salsuginea*, previously named *Thellungiella halophila*, separated from Arabidopsis around 15–30 million years ago and occupies an intermediate position between the *Brassica* and Arabidopsis clades [1-3]. Whereas this crucifer exhibits tolerance to high salt, drought, and cold stress, its close relative *Arabidopsis thaliana* is sensitive to these stresses. Comparative physiology and molecular biology studies demonstrated that different strategies are employed by these two species to adapt to stress conditions. *T. salsuginea* accumulates less sodium under salt stress than does Arabidopsis by using different ion uptake and translocation strategies. In contrast to Arabidopsis, the concentrations of sodium and potassium are not negatively correlated in *T. salsuginea*. Under salt stress, *T. salsuginea* plants undergo relatively high rates of transpiration and thus maintain water uptake and ion transport, which are crucial for plant survival [4]. The accumulation of high concentrations of compatible osmolytes in the cytosol, including sugars, polyols, and amino acids, is considered to be an effective strategy for acquiring tolerance to salt stress [5]. *T. salsuginea* contains higher levels of proline than does Arabidopsis under unstressed conditions, and accumulates 19-fold more proline in the shoot than does Arabidopsis under salt stress [6]. Microarray, qRT-PCR, and metabolite profiling analyses defined distinct stress-responsive genes and pathways that were active during the adaptation to stress conditions in *T. salsuginea* and Arabidopsis [3,4,7]. The expression patterns of many transcription factors diverged between these two species [3]. Given that the target genes of microRNAs (miRNAs) are predominantly transcription factors [8], we thought that it would be interesting to investigate the role of miRNAs in the regulation of salt tolerance in *T. salsuginea*.

Gene regulation by small RNAs represents a recently discovered layer of gene expression regulation in eukaryotes, and miRNAs are the best characterized of the small RNAs in plants [9]. miRNAs are ~21 nt non-coding RNAs that negatively regulate gene expression by mediating mRNA cleavage or translational inhibition of its target mRNA. Mature miRNA is preferentially incorporated into the RNA-Induced Silencing Complex (RISC) to direct the post-transcriptional repression of target mRNAs [9-11]. Previous studies demonstrated that miRNA regulates various aspects of plant growth and development. For example, miR165/miR166 is a critical regulator of adaxial/abaxial patterning in developing leaves [12], miR172 governs floral organ development [13,14], and miR159/miR319 regulates the level of TEOSINTE BRANCHED/CYCLOIDEA/PCF (TCP)-family transcripts, which control leaf development [15]. Increasing data from Arabidopsis, *Oryza sativa* (rice), and *Populus* indicate that miRNAs are stress-regulated and may play critical roles in the plant’s adaptation to environmental stress [11,16-19]. However, the roles of miRNAs in the extreme stress tolerance exhibited by the halophyte *T. salsuginea* have not been investigated. The release of the entire genome sequence of *T. salsuginea*[20,21] has greatly stimulated functional and comparative genomics studies of this species, and has enabled us to perform whole genome identification of *T. salsuginea* miRNAs, to predict their target genes, and to investigate how the salt-stress response differs from that in Arabidopsis at the level of miRNA regulation.

Although *T. salsuginea* can withstand exposure to high concentrations of NaCl [7,22] or sudden osmotic shock (i.e., transfer from 500 mM to 0 mM NaCl), the germination of *T. salsuginea* seeds is hypersensitive to NaCl. Shoot succulence of *T. salsuginea* was affected and growth was inhibited by 200 mM NaCl [4,23]. A previous study showed that 150 and 250 mM NaCl represent a minor irritation and moderate stress, respectively, for *T. salsuginea*[3]. To identify *T. salsuginea* miRNAs and investigate their possible roles in salt tolerance, we constructed two small RNA libraries. One library, which was used as the control, was derived from the tissues of 40-d-old plants grown under non-stress conditions, and the other library was constructed using 40-d-old plants treated with 200 mM NaCl for 24 h. We identified 109 known miRNAs, 26 novel miRNAs, and 111 plausible novel miRNA candidates from *T. salsuginea*. Target gene analysis and an investigation of the patterns of miRNA accumulation revealed important information about miRNAs in *T. salsuginea* that may elucidate salt tolerance mechanisms in this halophyte plant and others.

## Results

### Comparison of sRNA populations in CL and TL

**Figure 1:**
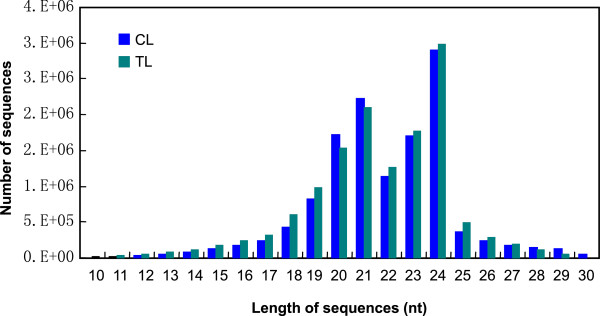
**Length distribution and abundance of small RNAs in the CL and TL.** CL: library constructed using plants grown under non-stressed condition; TL: library constructed using plants treated with 200 mM NaCl for 24 h.

To identify miRNAs in *T. salsuginea*, we constructed two small RNA libraries. The control library (CL) was constructed using plants grown in Hoagland solution without NaCl, and the treatment library (TL) used plants treated with 200 mM NaCl for 24 h. Previous studies indicated that the shoot succulence of *T. salsuginea* was affected and the growth was inhibited by 200 mM NaCl [4,23]. Total RNA was isolated from the roots and leaves 40 d after germination. Solexa technology (Illumina, Shenzheng, China) was used to sequence the small RNA enriched libraries, and 12,932,436 and 13,608,695 raw reads were generated from the CL and TL, respectively (Additional file 1: Table S1). The length of the small RNA sequences ranged from 10–30 nt (Figure 1). In both libraries, 24-nt sequences represented the largest group, followed by a group of 21 nt. Sequences of 20 nt and 23 nt represented the third largest group. The abundance of most small RNAs from 10–30 nt in the TL was higher than that in the CL. Only 20, 21, 28, 29, and 30-nt small RNAs were less abundant in the TL than in the CL (Figure 1).

After removal of the adaptor sequences, polyA sequences, and sequences of less than 18 nt, 12,010,658 and 12,330,771 clean reads were obtained from the CL and TL, respectively, and used for further analysis and miRNA discovery (Additional file 1: Table S1). The two libraries shared 831,074 unique small RNAs, which accounted for 14.16% of the total unique sequences. We detected 2,593,871 (44.18%) and 2,446,118 (41.66%) unique sequences in the CL and TL, respectively. Together, the two libraries shared 76.04% (18,509,850 out of 24,341,429) of the total small RNA sequences. We identified 3,060,900 (12.57%) and 2770679 (11.38%) small RNAs that were specifically present in the CL and TL, respectively (Additional file 2: Table S2). About 60% of these sequences could be successfully mapped to a particular region of the *T. salsuginea* genome (Additional file 3: Table S3).

Based on a comparison with the reference genome and relevant bioinformatics analysis, these small RNAs fell into the following categories: rRNA, tRNA, snRNA, exon_antisense, exon_sense, intron_antisense, intron_sense, unannotated, and miRNA candidate sequences. The miRNA candidate sequences represented only about 1.4% of the unique small RNAs (Additional file 4: Table S4). Most sequences (85% of the total unique sequences in both libraries) were unannotated (Additional file 4: Table S4). Although the total number of candidate miRNA sequences from the TL (1502495) was less than that from the CL (1904244), more clean reads were sequenced from the TL. Similarly, fewer unique candidate miRNA sequences were identified in the TL (47015) than in the CL (47793) (Additional file 3: Table S3 and Additional file 4: Table S4). These results indicate that miRNA biogenesis is down regulated in *T. salsuginea* when the plants are treated with 200 mM NaCl.

### Identification of known miRNAs and their variants

To identify miRNAs in *T. salsuginea* that have previously been reported in other plant species, small RNAs were compared with published miRNAs by conducting a Blastn search against miRbase 19.0 (April, 2013), in which 5141 plant miRNAs were registered. We identified 109 known miRNAs belonging to 23 conserved miRNA families and 9 known miRNA families (Table 1, Additional file 5: Table S5). The pre-miRNAs of 70 of these known miRNAs were discovered, and the secondary structures of these miRNAs are listed in Additional file 6: Figure S1. Previous studies indicated that 24 conserved miRNA families are present in both moncot and dicot model species [11,24]. miR397 is the only conserved miRNA family that was not detected in these two *T. salsuginea* libraries. We only considered sequences that perfectly matched previously identified mature miRNA sequences as known miRNAs. Sequences with one or two mismatches or gaps relative to the known miRNAs and that were represented in a minimum of 10 reads in at least one library were considered as miRNA variants (Additional file 7: Table S6). We identified a total of 1022 miRNA variants, which we named tsa-miRv1 to tsa-miRv1022. Many variants of some miRNA families were identified, highlighting the complexity of the miRNA variants. For example, we identified 395 variants of miR156.

**Table 1:** **Known miRNA families identified in *Thellungiella***

**miRNA families**	**Counts in CL**	**Counts in TL**
**Conserved miRNA families**		
miR156/157	1228075	784603
miR158	12	9
miR159	2831	2069
miR160	2898	2177
miR161	8063	10033
miR162	6564	4832
miR164	9708	10282
miR165/166	25193	30667
miR167	84175	78503
miR168	13427	19769
miR169	1819	1135
miR171	520	376
miR172	15420	14707
miR319	8	3
miR390	1107	854
miR391	4	7
miR393	55	29
miR394	16	30
miR395	126	170
miR396	786	541
miR398	1	5
miR399	9	4
miR408	1164	5044
**Non-conserved miRNA families**		
miR2111	35	26
miR400	316	215
miR403	632	474
miR824	6577	4667
miR827	1428	1238
miR845	98	91
miR5139	14	25
miR4995	4	11
miR894	42	209

We identified the *Thellungiella* homolog of most miRNAs that were conserved in Arabidopsis, *Populus trichocarpa*, *Vitis vinifera*, *Oryza sativa,* and *Brassica napus*, including miR156a, miR157a, d, miR164a, miR166a, and miR167a (Additional file 5: Table S5). A few miRNAs, such as miR824, miR827, and miR845, were only present in one to three of the five species. miR5139 and miR4995 were not present in these five species, while miR894 was only reported in *Populus trichocarpa* (Additional file 5: Table S5). In agreement with previous bioinformatics studies, these results indicate the varied extent to which different miRNA families are conserved [25]. In most cases, multiple members from one conserved miRNA family were detected; for example, we discovered 20 different members of miR156/157 and 13 different members of miR165/miR166. Interestingly, some miRNAs, including miR158, miR319, and miR398, were scarce, while their variants were abundant. For example, miR158 was sequenced about 20 times, while its variants (108 members) were sequenced more than 130,000 times in these two libraries. tsa-miRv396 and tsa-miRv397, the two variants of miR158, were the most abundant variants in *T. salsuginea*, being detected 54,501 and 24,028 times in CL and 31,356 and 16,098 times in the TL, respectively (Additional file 7: Table S6). These results demonstrate that tsa-miRv396 and tsa-miRv397 are the dominant mature sequences of miR158 in *T. salsuginea.*

**Figure 2:**
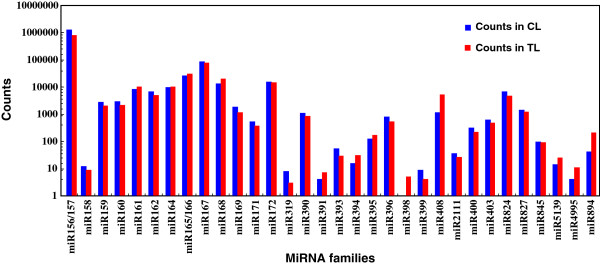
**The abundance of known miRNA families in *Thellungiella*.** Abundance is represented by the number of reads per ten million (TP10M).

The abundance of identified conserved miRNAs was significantly different, ranging from only a few to more than half a million counts. The most abundant expressed miRNA in *T. salsuginea* was miR156a, which was detected 665,846 and 394,884 times in the CL and TL, respectively (Figure 2, Additional file 5: Table S5). miR156q, miR157a, miR157d, miR166a, miR167a, miR168a, and miR172a were highly expressed, with more than 10,000 reads sequenced in each library. About 10 other miRNA members showed a moderate expression level, with 1,000 to 9,000 reads in each library. The abundance of most other miRNAs (> 80%) was low, ranging from a few to a hundred counts in each library (Figure 2, Additional file 5: Table S5.

A comparison of the 109 known miRNAs in the two libraries revealed that the abundance of 26 miRNAs decreased under salt treatment (ratio ≤ 0.67), including three miRNAs detected only in the CL. Only 17 miRNAs exhibited more than a 1.5-fold upregulation upon 200 mM NaCl treatment. However, the abundance of some miRNAs was extremely low in both the CL and TL, suggesting that the differential expression of these miRNAs may not be linked to the salt response. Overall, the abundance of miRNAs that exhibited increased expression in the TL was low. However, miR161, miR165a, miR166a, and miR168a were strongly expressed in both libraries and were up-regulated in the TL (Figure 2, Additional file 5: Table S5). The abundance of 380 variants was down-regulated (ratio ≤ 0.67), including that of 7 variants detected only in the CL. The NaCl treatment up-regulated the expression of 220 variants by more than 1.5-fold, including 4 variants detected only in the TL. The expression patterns of most variants were in accordance with those of known miRNAs (Additional file 7: Table S6). *T. salsuginea* miR894 and its variants tsa-miRv977 and tsa-miRv978 were highly expressed in both libraries, particularly after the NaCl treatment. *T. salsuginea* miR408 was strongly induced by the NaCl treatment, while Arabidopsis miR408 was induced by cold and mannitol, but not by salt [19]. The differential response of these miRNAs to salt stress suggests that they may have a key role in salt tolerance.

### Identification of novel miRNAs

We used previously described criteria to predict specific miRNAs of *T. salsuginea*[26,27]. The precursor of each novel miRNA was identified and could form a proper secondary hairpin structure with a maximal free energy of -18 kcal/mol. We identified 26 novel miRNA families and 111 plausible candidate miRNAs (PCs) from the two libraries (Table 2, Additional file 8: Figure S2 and Additional file 7: Table S7). The free energy of the identified novel miRNAs and PCs ranged from -18.3 kcal mol^-1^ to -173.5.kcal mol^-1^. Blastn searches against all nucleotide sequences in NCBI databases were performed using these miRNA sequences as queries. No homolog was found in any other plant species, suggesting that these newly identified candidate miRNAs are all *Thellungiella*-specific. The abundance of most miR* sequences identified in this study was less than 10 counts, except for tsa-miRn3, tsa-miRn8, tsa-miRn11, and tsa-miRn20 (Table 2). Previous studies indicated that both the 5p and 3p sequences of some miRNAs could be recruited into the silencing complex and function as miRNAs [28]. The abundance of the majority of novel miRNAs and plausible candidates was relatively low compared with that of conserved miRNAs. However, some novel miRNAs were abundant; for example, miRn13 (3777 counts in the CL, 2846 counts in the TL), tsa-miRn20-3p (639 counts in the CL, 523 counts in the TL), miRn7 (1927 counts in the CL), PC003 (397 counts in the CL, 652 counts in the TL), and PC070 (646 counts in the CL, 3201 counts in the TL) (Table 2, Additional file 7: Table S7).

**Table 2:** **Novel miRNAs identified in *Thellungiella***

**Novel miRNA**	**Novel miRNA sequence**	**Length**	**miRNA count**		**Genomic location of pre-miRNA**	**MFE**	miR*	miR* count	
			**CL**	**TL**				**CL**	**TL**
tsa-miRn1	GGAGGAUGAUACAAGCUCUCAUA	23	24	0	scaffold_10:7369671:7369761:+	-18.5	CUUCAACAUUAGAAUGCAUGUAUC	1	0
tsa-miRn2	UAGGAAUCUGUGCUCAAACCAC	22	18	22	scaffold_11:1867622:1867724:+	-57.1	UUGAGCACAGAUUUCUACA	1	1
tsa-miRn3-5p	UUGGGGGUAAGAGUAUUAUAC	21	78	72	scaffold_13:6097671:6097798:-	-46.1	UAUAAUACCCUUAAACCCAAU	10	10
tsa-miRn3-3p	UAUAAUACCCUUAAACCCAAU	21	10	10			UUGGGGGUAAGAGUAUUAUAC	78	72
tsa-miRn4	UGUUCGCCGGAAAAUAACCAG	21	47	55	scaffold_15:2449702:2449784:+	-48.2	GGUUAUUUUCCGGCGAAGCAGA	1	0
tsa-miRn5	UUUUAAAACUGAAAACGUAAU	21	92	77	scaffold_15:2733677:2733793:+	-45.3	UACGUUAUCAGUUUUAAAACA	1	1
tsa-miRn6	AUUGGAGUUGAUAUAAUGCAG	21	0	10	scaffold_9:4431521:4431709:+	-71.2	CAACCUGCAUUAUAUCAACUC	0	1
tsa-miRn7	CAAGGCAGAAGAAGGCUGUUU	21	1927	0	scaffold_19:2418388:2418480:+	-63.1	ACAGCCUCUUCUUCCUUGUU	1	0
tsa-miRn8-5p	AAUGAAUGAUGCGGUAGACAAAU	23	123	71	scaffold_1:6999873:6999998:-	-36	UAGCUUCCGACUCAUUCAUCCA	13	9
tsa-miRn8-3p	UAGCUUCCGACUCAUUCAUCCA	22	13	9			AAUGAAUGAUGCGGUAGACAAAU	123	71
tsa-miRn9	AGGAGAGUUUUGGUGUAGCAA	21	21	23	scaffold_229:434:568:-	-30.7	UUCUAUGCUCAGAACUCCUUCUU	0	1
tsa-miRn10	UGAAUUUGAUUUUAGACAGGA	21	26	0	scaffold_2:7409777:7409908:+	-57.1	UGUCUGAAAUCAGGUUCAGGUA	1	0
tsa-miRn11-3p	UGAGUCGUCAAUCAGUAAGGU	21	228	247	scaffold_2:8810421:8810518:+	-34.9	AUUACUUGUUGACGAUUCCUU	252	133
tsa-miRn11-5p	AUUACUUGUUGACGAUUCCUU	21	252	133	scaffold_2:8810421:8810518:+		UGAGUCGUCAAUCAGUAAGGU	228	247
tsa-miRn12	UGAUAGCAGUAGUUUCGUCUA	21	192	172	scaffold_2:856191:856302:-	-23.5	AGAGAUCGGAGCUUUGUUGG	1	0
tsa-miRn13	UAGUGAAAUUGGAAAGUUGCC	21	3777	2846	scaffold_2:6151436:6151550:-	-49	AACGACAGCUUUCCUGAUUCCA	2	1
tsa-miRn14	UCAUGAAGGAUCUGAGAUUGA	21	78	0	scaffold_2:18154827:18154925:-	-48.1	CUCCAGUCUCAUACUCUUCAU	2	0
tsa-miRn15	CUAGGGUUCCAGAAUUGAGGCUA	23	57	32	scaffold_3:8786082:8786170:+	-26.4	GCUUUAAUUCGGAGUUGGUCGGC	1	1
tsa-miRn16	UGUGAUCCUCAGAUAGACGUACA	23	4	10	scaffold_3:10218595:10218739:+	-38.3	AGUUGUCAUUAUAUCUCUGAUCGC	0	2
tsa-miRn17	ACCCCAAACCAGCUCAGACAA	21	142	263	scaffold_3:2561950:2562105:-	-67.7	CAGCUUAUUUGGGAUUGGAGGUC	0	3
tsa-miRn18	UGGAUUUAGAAUAAUGGUGGCUA	23	127	118	scaffold_4:1893254:1893541:+	-47.63	AUCAUUGCUCUGAUACCAUGUUGA	2	3
tsa-miRn19	AAGGCUGUGAAUUGUUUUGGC	21	167	116	scaffold_7:7785014:7785113:-	-57.26	CGAAACAAUUCACAGUCUUGA	0	1
tsa-miRn20-3p	AAAGCAUGUGAGUACUUCGUA	21	639	523	scaffold_9:9328524:9328666:-	-71.2	UGAAAUAUUCACAUGCUUUCG	357	439
					scaffold_9:9328524:9328666:-	-71.2			
tsa-miRn20-5p	UGAAAUAUUCACAUGCUUUCG	21	357	439			AAAGCAUGUGAGUACUUCGUA	639	523
tsa-miRn21	UGAAAGGAAACAUUGAUGUUU	21	0	82	scaffold_15:1338317:1338445:-	-50.1	ACAACAGUGUUUUCUUUCACA	0	9
tsa-miRn22	UUGUGCAAGACUGAGAAGCAA	21	0	10	scaffold_5:15200075:15200174:+	-57.5	UCUUCUUCCUCUUGCACAACC	0	1

Novel miRNA is the name of detected novel miRNAs. -5p and -3p means the novel miRNA and miR*. Genomic locations of pre-miRNA are listed: The *T. salsuginea* genome sequence was downloaded from the *T. salsuginea* Sequencing Resource: website ftp://ftp.jgi-psf.org/pub/compgen/phytozome/v9.0/Thalophila/assembly/). +, sense strand. -, anti-sense strand.

Many miRNAs were detected in only one library; for instance, tsa-miRn6, tsa-miRn21, and tsa-miRn22 were only present in the TL, and tsa-miRn1, tsa-miRn7, tsa-miRn10, and tsa-miRn14 only in the CL (Table 2). Thirty-one novel candidate miRNAs were only detected in the CL and thirty were only found in the TL (Additional file 7: Table S7). Due to the low abundance of most novel miRNAs, the specificity of these miRNAs in one library may not truly reflect a specific expression pattern under a particular growth condition. However, a few putative miRNAs were expressed only in one library at relatively high abundance (Table 2, Additional file 7: Table S7). These miRNAs may function as key regulators of the salt stress response in *T. salsuginea.* Previous functional studies of the predicted target genes of tsa-miRn7 and PC069 support this hypothesis [29,30] (Additional file 7: Table S8). Considering the environmental, developmental, and tissue-specific regulation of miRNA expression, the miRNAs identified in our study probably represent only part of the *T. salsuginea* miRNA population. It remains to be determined how much overlap exists between the miRNAs identified in this study and those that are responsive to cold or drought stress.

### Expression analysis of miRNAs by microarray and qRT-PCR analysis

**Figure 3:**
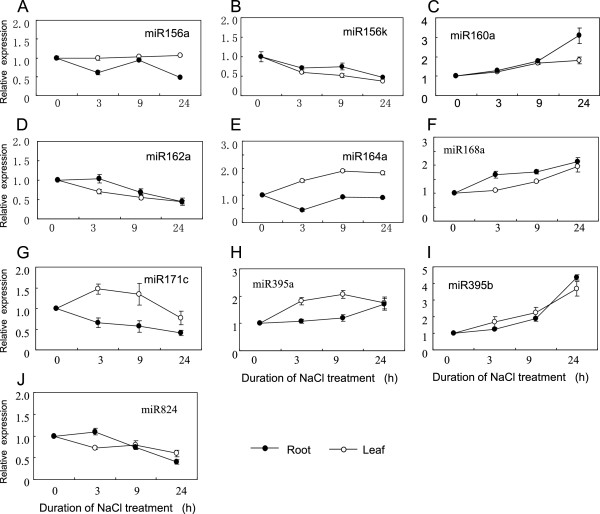
**miRNA expression analysis using Affymetrix chip.** RNA samples were isolated from *Thellungiella* roots and leaves at 0 h, 3 h, 9 h, and 24 h treated with 200 mM NaCl. Bars represent the standard deviations of three replicates. **(A)** miR156a. **(B)** miR156k. **(C)** miR160a. **(D)** miR162a. **(E)** miR64a. **(F)** miR168a. **(G)** miR171c. **(H)** miR395a. **(I)** miR395b. **(J)** miR824. Duration of NaCl treatment **(h)**.

The expression of some conserved miRNAs in leaves and roots was examined in the presence or absence of NaCl treatment using an Affymetrix miRNA chip. About 400 probes on this chip corresponded with 45 plant miRNAs. These probes (miRNAs) shared identical sequences with the *Thellungella* miRNAs. Twenty-six of these miRNAs were represented by more than three probes on the chip and were used in the expression analysis. The remaining 19 miRNAs, which had less than three probes on the chip, were not used in the expression analysis. In this study, leaf and root tissues were sampled separately after 3, 9, and 24 h of NaCl treatment. The expression of some conserved miRNAs was up- or down-regulated (1.5-fold) by the NaCl treatment (Figure 3). Differential expression patterns in the roots and leaves were observed for some miRNAs (Figure 3). For example, the expression of tsa-miR164a and tsa-miR171c decreased in roots, but increased in leaves after salt treatment.

**Figure 4:**
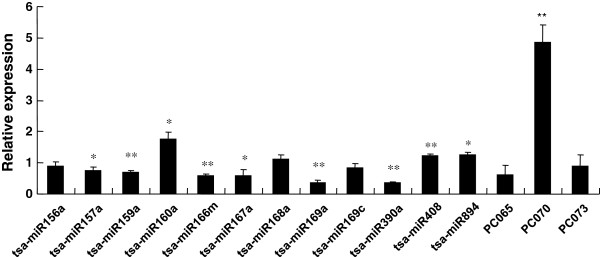
**Relative expression of miRNAs as determined by stem-loop qRT-PCR.** Total RNA isolated from control seedlings and those treated with 200 mM NaCl. The reverse transcription reaction was carried out using stem-loop primers (Additional file 9: Table S9) of these miRNAs. The level of expression was normalized to that of U6. The normalized miRNA levels in the control were arbitrarily set to 1. Error bars indicate ± SE obtained from three biological repeats. Student’s *T*-test was performed to analyze the changes in the gene expression after treated with NaCl. **denotes the p value < 0.01 and *denotes the p value < 0.05.

To verify the expression of the newly identified *Thellungella* miRNAs, a stem-loop qRT-PCR assay was carried out. The expression of nine conserved miRNAs and two candidate novel miRNAs (PC065 and PC073) was analyzed. The results were consistent with the Solexa sequencing data (Figure 4).

### Target gene prediction of known and novel miRNAs

**Figure 5:**
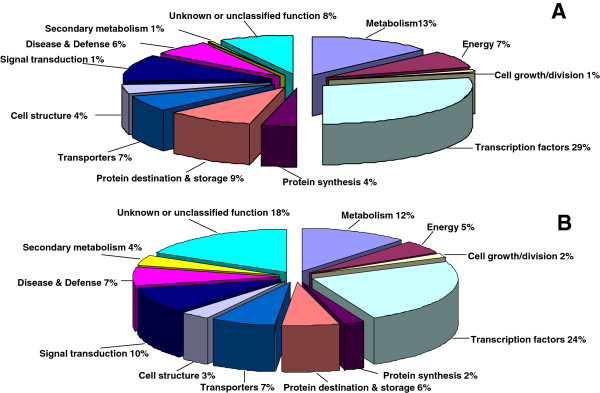
**Function classification of the target genes of known and novel miRNAs in *Thellungiella*. A**: Target genes of known miRNA, **B**: Target genes of novel and pc miRNA.

We predicted more than 143 potential target genes for all of the 32 known *T. salsuginea* miRNA families. These target genes included 42 (29%) transcription factors (Figure 5A, Additional file 7: Table S10). Interestingly, three stress-responsive transcription factors, one ABA-responsive factor, and two ethylene-responsive factors were the predicted targets of *Thellungiella* miRNAs. Genes involved in a broad range of developmental and physiological processes were also predicted to be miRNA targets in this study. Several genes encoding ion transporters or ion channels were identified as target genes of *Thellungiella* miRNAs, including an inward rectifying potassium channel, high-affinity nickel-transport family protein, Ca^2+^ transporting ATPase, plasma membrane sulphate transporter, boron transporter, calcium-binding protein annexin 7, Fe(II) transport protein IRT1, putative cadmium/zinc-transporting ATPase 3, and sulfite exporter TauE. In addition, many target genes identified in this study were directly or indirectly involved in the stress response or stress tolerance, including two salt-inducible proteins and two E3 ubiquitin ligases [31].

Following the published criteria for target gene prediction [8], 425 target genes were predicted from 26 novel and 111 PC *Thellungilla* miRNAs. These predicted target genes had a wide range of biological functions, and many encode transcription factors. One-hundred-and-nine targets encoded transcription factors or splicing factors thought to affect gene expression directly. Many of these transcription factors (e.g., auxin response factor, no apical meristem (NAM) protein, NAC transcription factor, and WRKY transcription factor) are key regulators in the salt, drought, or cold stress response [32-35]. Interestingly, ion and macromolecular transporters, which may play key roles in ion homeostasis or the accumulation of compatible molecules under stress conditions, were significantly enriched in targets of these novel miRNAs. Many miRNAs were predicted to target a variety of transporters and ion channels, such as an anion transporter, ion channel CLC-3, ABC transporter, and efflux pump (MatE). Furthermore, a number of other proteins known to be key players in the stress response were predicted to be targets of the *Thellungiella* novel miRNAs or miRNA candidates (Figure 5, Additional file 7: Table S8). The discovery that genes directly or indirectly involved in stress response pathways are potential targets of the newly identified *Thellungiella* miRNAs suggests that miRNA regulation is an important layer of stress tolerance in this plant.

### Validating the cleavage of target mRNA by 5’ RACE

**Figure 6:**
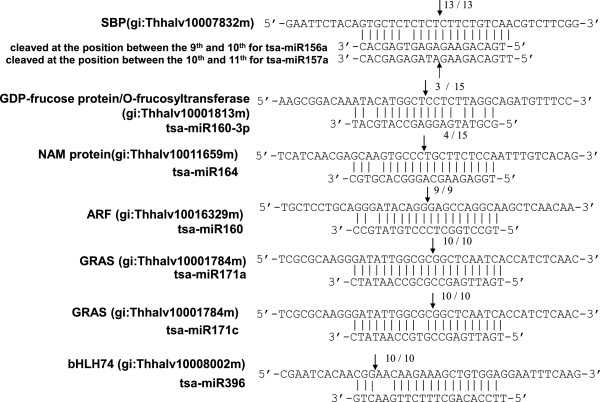
**Verification of target gene cleavage sites by 5’ RACE.** For each miRNA, the partial target sequence is shown at the top and the miRNA sequence at the bottom. Perfectly complementary bases are connected by solid lines. Arrows indicate cleavage sites. Numbers indicate the fraction of cloned PCR products terminating at the cleavage site.

To test whether the identified miRNAs could mediate the cleavage of the predicted target mRNAs, a 5’ RACE experiment was performed. The targets of the conserved miRNAs, miR156/157, miR160, miR160-3p, miR164, miR171a, and miR171c, were cleaved at a site corresponding to the 10th miRNA nucleotide from the 5’ end (Figure 6). Mismatches between the miRNA molecules and their binding sites on the target were rare. miR171a was 100% complementary to the binding site of the target. One or two mismatches were indentified in the other miRNA/target pairs. No mismatches were found at the 10th and 11th nucleotides in any of these miRNA/target pairs.

Due to high levels of sequence similarity, the different members within an miRNA family may target the same gene. For example, several miR156/157 members could target the SBP transcription factor at the same site on the target mRNA. We analyzed an SBP gene with high sequence similarity to Arabidopsis SPL10 by 5’ RACE. The target was cleaved at the position between the 9th and 10th nucleotides by *Thellungiella* miR156a, miR156e, and miR157b. However, this position was corresponding to the 10th and 11th nucleotide of *Thellungiella* miR156n, miR156o, and miR157a (Figure 6, Table 1, Additional file 5: Table S5). In *Phaseolus vulgaris,* the cleavage site of the SBP-like related gene was located between the 10th and 11th nucleotide of miR156a [36]. An SBP transcription factor, a PPR repeat protein, and a MADS box transcription factor were predicted to be targets of tsa-miR156/157, tsa-miR158, and tsa-miR824, respectively. However, the 5’ RACE experiment only detected cleavage of these genes upstream or downstream of the miRNA/target-binding region, but not at the predicted sites (data not shown). Thus, the target cleavage positions were predominantly at around the 10th nucleotide from the 5’ end of the miRNA, but some might mediate cleavage at other locations.

## Discussion

Accumulating evidence suggests that miRNAs play essential roles in both abiotic and biotic stress responses. However, a systematic identification of miRNAs from the stress-tolerant model plant *T. salsuginea* had hitherto not been accomplished. Taking advantage of the sequenced genome of *T. salsuginea* and high-throughput sequencing technology, the present study reported the first large-scale identification of *T. salsuginea* miRNAs. A total of 109 known miRNAs belonging to 23 conserved miRNA families and 9 non-conserved known miRNA families, as well as 26 novel miRNAs and 111 plausible candidates were discovered. Some of the miRNAs were differentially expressed in the presence or absence of 200 mM NaCl. Interestingly, three novel and 30 plausible candidates of novel miRNAs were only detected in NaCl-treated samples.

Most of the conserved miRNAs exhibited decreased abundance upon treatment with 200 mM NaCl. Only 17 miRNAs showed more than a 1.5-fold increase in abundance after NaCl treatment. The overall abundance of these differentially expressed miRNAs was low in both libraries, with the exception of miR166f, miR168a, tsa-miR408, and tsa-miR408-5p. The abundance of these miRNAs was high in both libraries. In agreement with our results, previous studies consistently demonstrated that miR166, miR168a, and miR408 were all responsive to stress conditions, including salt and drought stress [19,37-40].

The expression of miR389 was inhibited and the expression of miR397 and miR402 was induced by salt, cold, dehydration, and ABA in Arabidopsis [16], while these three miRNAs and their variants were not detected in *T. salsuginea. Phaseolus* miR393 was induced by NaCl, dehydration, and ABA, but *Thellungiella* miR393a was only detected once in each library, and miR393b-3p showed decreased expression upon salt treatment. In agreement with results from *Phaseolus*[36], *Thellungiella* miR159b exhibited increased expression levels under salt stress (Additional file 5: Table S5). MiR167c was strongly up-regulated by 24 h of NaCl treatment in Arabidopsis, while *Thellungiella* miR167a-h showed similar or decreased expression levels under NaCl stress conditions [19] (Additional file 5: Table S5). miR398 expression was induced by salt treatment in Populus, while it was repressed in Arabidopsis [41]. Very low levels of miR398 expression were detected in both control and NaCl-treated *Thellungiella* libraries (1 vs. 5); however, its variant, tsa-miRv878, was sequenced with relatively high abundance (98 vs. 237), indicating that NaCl induced miR398 in *Thellungiella.* The conservation and divergence of miRNA expression under different stress conditions illustrates the complexity of miRNA regulation.

The target genes of all identified known miRNA families, 26 novel miRNAs, and 109 plausible candidates were predicted. The identified target genes are involved in multiple biological processes. Many transport proteins or ion channels were predicted as targets of conserved and *Thellungiella-*specific miRNAs. Some of these proteins have been well studied and their roles in salt tolerance or the stress response have been established. Maintenance of Na^+^, K^+^ homeostasis is required for plant survival in a high salinity environment. HKT1, a K^+^ transporter, contributed significantly to Na^+^ uptake in Arabidopsis and rice and plays key roles in salt tolerance [42-44]. Substantial transcriptional regulation of the high affinity K^+^ transporter was observed under salt stress [45]. The K^+^ transporter gene was identified as a novel miRNA target in *Thellungiella*. An inward rectifying potassium channel, a member of the AKT protein family, exhibits non-specific Na^+^ uptake activity under high concentrations of Na^+^[46]. In the halophytic plant *M. crystallinum,* the expression of the AKT1 homolog decreased in response to salt stress [47]. We found that the inward rectifying potassium channel was the target of *Thellungiella* miRNA.

Besides tolerance to salinity, *Thellungiella* exhibits high tolerance to dehydration and cold stresses [23,48-51]. The novel miRNA candidates identified in our study may represent small RNAs that are expressed in a relatively high level under non-stress or salt stress conditions. A previous study revealed that gene expression in response to cold, drought, and salinity showed little overlap [49]. Therefore, we do not expect much overlap in *Thellungiella* miRNAs involved in the plant’s response to different stresses. It would be interesting to determine which miRNAs from *Thellungiella* are activated upon exposure to cold or drought stresses.

## Conclusions

About thirteen million high-quality small RNAs were sequenced from each library. Totally, we identified 109 known miRNAs and 137 novel miRNA candidates from *T. salsuginea*. In addition, 568 target mRNAs were predicted. Differentially expressed miRNAs between NaCl treated and the control samples were analyzed using Solexa sequence data and part of them were validated by microarray and together with qRT-PCR. This study lay the basis for functional studies of miRNA in *T. salsuginea* and enables us to access the salt tolerance mechanism of this plant species from the layer of small RNA regulation.

## Methods

### Plant materials

*Thellungiella salsuginea* (Shandong ecotype) seeds were surface-sterilized, sown on 1/2 MS agar plates, stratified at 4°C for one week, and then moved to a growth chamber for one week at 26°C/22°C (day/night) and with 16 h of illumination per day. Seedlings were transferred to a planting box containing Hoagland solution, which was placed at a relative humidity of 75% and 26/20°C day/night temperature with a light intensity of 3000 lx. Forty-day-old plants treated with 200 mM NaCl for 24 h and non-treated plants were used to prepare small RNA libraries.

### Small RNA library construction and sequencing

Total RNA was extracted from *T. salsuginea* using Trizol Reagent (Invitrogen, USA), following the manufacturer’s protocol. RNA quality was evaluated by electrophoresis on a 1% agarose gel. RNA concentration was quantified using BioSpectrometer fluorescence (Eppendorf, GER). Two small RNA libraries of *T. salsuginea* were generated and were sequenced by Solexa technology. The sequencing procedure was conducted as described in our previous work [52]. Briefly, after PAGE purification of small RNA molecules and ligation of a pair of Solexa adaptors to their 5' and 3' ends, the small RNAs were amplified for 17 cycles using the adaptor primers, for cluster generation and sequencing analysis. The image files generated by the sequencer were then processed to produce digital-quality data by the BGI small RNA pipeline. After masking the adaptor sequences and removing contaminated reads, clean reads were processed for computational analysis. A computational pipeline was developed to process the sequencing data and to distinguish miRNA candidates from other types of small RNAs.

### Data analysis

The raw reads were processed using PHRED and CROSS MATCH. Vector sequences, low quality sequences, and sequences that are shorter than 10 nt were removed. The clean reads were used for further analysis. Small RNA tags were mapped to the *T. salsuginea* genomes using the SOAP program [53]. rRNA, scRNA, snoRNA, snRNA, and tRNA were removed by conducting a BlastN search against GenBank and Rfam. The remaining small RNAs were aligned with exons and introns of genes to identify degraded mRNA fragments.

The *T. salsuginea* genome sequence was downloaded from the *T. salsuginea* Sequencing Resource website (http://www.phytozome.net/thellungiella.php; *T. salsuginea* genome reference sequence ftp://ftp.jgi-psf.org/pub/compgen/phytozome/v9.0/Thalophila/assembly/). Small RNAs were mapped to the genome using PatMaN. The hairpin structure was predicted using online software mfold (http://www.bioinfo.rpi.edu/applications/mfold/). MiRNA candidates were predicted using miRCat (http://srna-tools.cmp.uea.ac.uk/plant) [54] with default parameters. *T. salsuginea* transcript reference sequences (ftp://ftp.jgi-psf.org/pub/compgen/phytozome/v9.0/Thalophila/annotation/ annotation/Thalophila_173_transcript.fa.gz) and NCBI dbEST (http://www.ncbi.nlm.nih.gov/nucest/?term=Thellungiella) were used for the target prediction. The number of small RNAs was normalized by TP10M (tags per ten million), which represents the abundance of small RNAs. The TL/CL ratio was used to calculate the fold changes in expression level of known and novel miRNAs.

### Identification of known miRNAs and their variants

The mature miRNAs of miRBase 19.0 (http://www.mirbase.org/) were downloaded and aligned with small RNA tags by BlastN: blastall-p blastn-F F -e 0.01. Perfectly matched sequences were considered as known miRNAs in *T. salsuginea,* and the other sequences, with no more than two mismatches or gaps, were considered as variants. In addition, the abundance of the variant was no less than 10 counts in at least one miRNA library. The known miRNAs* were predicted in the hairpin structures using Mireap software.

Up to April 2013, 25141 mature miRNAs from 193 species are available in miRBase 19.0. An miRNA gene always has different variants due to imprecise processing or various biochemical modifications, and the miRNA annotation is defined by the dominant sequence if it is not the same as sequences in miRBase. Deep sequencing could detect miRNA* sequences that are too rare to be cloned by traditional cloning and sequencing methods. MiRNA genes show a characteristic “mapping pattern”, which is the result of sequential processing of Drosha and Dicer. According to this feature, false miRNAs can be removed from the known small RNA dataset by manual check. The number of reads sequenced was used to measure the abundance of a specific miRNA species in the library. Due to inaccurate processing and base modification, miRNA always has more than one sequence form, and the sum of all variants of an miRNA gene could be used as a digital measure of its expression.

### Identification of novel miRNAs and plausible candidates of novel miRNAs

Novel miRNAs and plausible candidates of novel miRNAs were predicted based on the hairpin structure of miRNA precursors using Mireap software with the following parameters: dominant mature sequences reside at the stem region with a size of 18–25 nt; the abundance is at least ten counts; the precursor of each novel miRNA was identified and could form a proper secondary hairpin structure; and the distance between miRNA and miRNA* is 16–300 nt. The flank sequence length of the miRNA precursor is 20 nt and the maximal free energy allowed to form a hairpin structure is -18 kcal/mol. miRNAs were considered novel if the miRNA* sequence was detected in at least one library. miRNAs for which the miRNA* was not detected in the sequenced libraries were considered as plausible novel miRNA candidates.

### Identification and functional annotation of the target genes

Target genes of conserved and novel miRNAs were predicted using perl software pick_plant_target.pl with the following parameters: (1) no more than four mismatches were permitted between the sRNA and target (G-U bases count as 0.5 mismatches); (2) no more than two adjacent mismatches in the miRNA/target duplex; (3) no adjacent mismatches in positions 2–12 of the miRNA/target duplex (5' of miRNA); (4) no mismatches in positions 10–11 of the miRNA/target duplex; (5) no more than 2.5 mismatches in positions 1–12 of the miRNA/target duplex (5' of miRNA); and (6) the minimum free energy (MFE) of the miRNA/target duplex should be ≥ 75% of the MFE of miRNA bound to its perfect complement. The coding sequences of predicted target genes were downloaded (http://www.phytozome.net/search.php?show=text&method=Org_Tsalsuginea, http://www.ncbi.nlm.nih.gov/nucest/?term=Thellungiella) and submitted to NCBI for BlastX analysis with an e-value of 1e-10 to identify putative gene homologs and the functions of potential targets.

### MiRNA expression profiling using microarray analysis

In Affymetrix genechip Sanger miRNA V11.0, about 400 probes corresponding to 45 unique plant miRNAs with sequences identical to *T. salsuginea* miRNA sequences. Total RNAs were extracted from leaves and roots using Trizol Reagent (Invitrogen, USA). One microgram of total RNA was used to isolate low molecular weight RNA using polyethylene glycol solution precipitation. Low molecular weight RNAs were labeled with Cy3 and hybridized with miRNA microarrays (Shanghai BioCorp., China). Arrays were scanned using a GeneChip®Scanner 3000. Hybridization signals were extracted from the TIFF images using GCOS1.4 software. miRNA QC Tool software was used for data summarization, normalization, and quality control. Low signal probes and probes with fewer than three replicates on the chip were removed.

### Detection of miRNA expression using stem-loop qRT-PCR

The stem-loop qRT-PCR primers (Additional file 9: Table S10) were designed and the reverse transcription was carried out according to previous studies [55,56]. Stem-loop qRT-PCR was performed in an ABI StepOne Real-Time PCR System (Applied Biosystems) using SYBR Green I (TOYOBO) with the following program: 94°C for 10 min, followed by 40 cycles of 94°C for 15 s, 60°C for 10 s, and 72°C for 25 s. All reactions were conducted in triplicate, U6 was used as an internal control, and the sample without template was used as a negative control. The relative expression of the miRNA was calculated using ABI StepOne Real-Time PCR System software and the 2^-ΔΔ^Ct method.

### Verification of the miRNA cleavage site by 5’ RACE

Total RNA was isolated from NaCl-treated and control plants using the CTAB method. Equal amounts of total RNA were pooled together and used for cDNA synthesis following the protocols of the SMARTer™ RACE cDNA Amplification Kit (Clontech). Gene-specific reverse nest primers were designed from the predicted targets and used in combination with the 5’ adapter primers (UPM, NUP) to amplify the cleaved transcripts. PCR reactions were separated by agarose gel electrophoresis, and distinct bands of the expected size were cloned into pMD18 simple-T vectors (Takara, Dalian, China) and sequenced using the general primer M13.

## Competing interests

The authors declare that they have no competing interests.

## Authors’ contributions

QZ, XW and YZ designed the study. QZ and CZ carried out most of the experiments, data analysis and wrote the material and method part of the manuscript. XW, JP and YZ wrote the manuscript, made the figures and finalized the tables. WS, HX, YL, AL, CL, ML, MS, SZ, LH, performed experiments and took care of the plants. All authors read and approved the final manuscript.

## Supplementary Material

Additional file 1: Table S1 Raw reads generated by Solexa sequencing. Click here for file

Additional file 2: Table S2 Common and specific small RNAs identified in the CL and TL. Click here for file

Additional file 3: Table S3 Small RNAs mapped in the *Thellungiella salsuginea* genome. Click here for file

Additional file 4: Table S4 Distribution of different small RNA categories in the CL and TL. Click here for file

Additional file 5: Table S5 Detailed information of known miRNAs identified in *Thellungiella salsuginea*. Click here for file

Additional file 6: Figure S1 Hairpin structures of known miRNAs of *Thellungiella*. Click here for file

Additional file 7: Table S6 MiRNA variants identified in *Thellungiella salsuginea*. **Table S7** Plausible candidates of novel miRNAs identified in *Thellungiella salsuginea.***Table S8** Targets of novel miRNAs in *Thellungiella salsuginea.***Table S10** Targets of known miRNAs in *Thellungiella salsuginea*. Click here for file

Additional file 8: Figure S2 Hairpin structures of novel miRNAs of *Thellungiella*. Click here for file

Additional file 9: Table S9 Stem-loop qRT-PCR primers used in the study of tsa-miRNA expression. Click here for file
