# Supplementary material for: Genome-wide identification of Thellungiella salsuginea microRNAs with putative roles in the salt stress response
Source: BMC Plant Biol. 2013 Nov 15;13:180. doi: 10.1186/1471-2229-13-180 (PMC4225614; doi:10.1186/1471-2229-13-180)
Supplement: Additional file 1: Table S1 — Raw reads generated by Solexa sequencing. [file 1471-2229-13-180-S1.doc]

**Table S1 Raw reads generated by Solexa sequencing**

| **read type** | **(CL) CK** | | **(TL) NaCl** | |
| --- | --- | --- | --- | --- |
|  | **count** | **percentage** | **count** | **percentage** |
| Raw reads | 12932436 | 100% | 13608695 | 100% |
| 3'adapter null | 20191 | 0.16% | 4875 | 0.04% |
| insert null | 8984 | 0.07% | 9829 | 0.07% |
| 5'adapter contaminants | 181351 | 1.40% | 247280 | 1.82% |
| smaller than 18nt | 708933 | 5.48% | 1014198 | 7.45% |
| polyA | 2319 | 0.02% | 1742 | 0.01% |
| **clean reads** | 12010658 | 92.87% | 12330771 | 90.61% |
