# Supplementary material for: Genome-wide identification of Thellungiella salsuginea microRNAs with putative roles in the salt stress response
Source: BMC Plant Biol. 2013 Nov 15;13:180. doi: 10.1186/1471-2229-13-180 (PMC4225614; doi:10.1186/1471-2229-13-180)
Supplement: Additional file 2: Table S2 — Common and specific small RNAs identified in the CL and TL. [file 1471-2229-13-180-S2.doc]

**Table S2 The common and specific small RNAs in CL and TL libraries**

| **Sequence types** | **No of unique sRNAs** | **%** | **No of total sRNAs** | **%** |
| --- | --- | --- | --- | --- |
| Total sRNAs | 5871063 | 100.00 | 24341429 | 100.00 |
| sRNAs shared by CL and TL | 831074 | 14.16 | 18509850 | 76.04 |
| sRNAs detected only in CL | 2593871 | 44.18 | 3060900 | 12.57 |
| sRNAs detected only in TL | 2446118 | 41.66 | 2770679 | 11.38 |
