# Supplementary material for: Genome-wide identification of Thellungiella salsuginea microRNAs with putative roles in the salt stress response
Source: BMC Plant Biol. 2013 Nov 15;13:180. doi: 10.1186/1471-2229-13-180 (PMC4225614; doi:10.1186/1471-2229-13-180)
Supplement: Additional file 3: Table S3 — Small RNAs mapped in the Thellungiella salsuginea genome. [file 1471-2229-13-180-S3.doc]

**Table S3 Small RNAs mapped in *Thellungiella salsuginea* genome**

| **Sequence type description** | **Unique sRNAs** | **%** | **Total sRNAs** | **%** |
| --- | --- | --- | --- | --- |
| CL derived sRNAs | 3424945 | 100% | 12010658 | 100% |
| CL sRNAs mapped to genome | 2008752 | 58.65% | 9118243 | 75.92% |
| TL derived sRNAs | 3277192 | 100% | 12330771 | 100% |
| TL sRNAs mapped to genome | 1983164 | 60.51% | 9816751 | 79.61% |
