# Supplementary material for: Genome-wide identification of Thellungiella salsuginea microRNAs with putative roles in the salt stress response
Source: BMC Plant Biol. 2013 Nov 15;13:180. doi: 10.1186/1471-2229-13-180 (PMC4225614; doi:10.1186/1471-2229-13-180)
Supplement: Additional file 4: Table S4 — Distribution of different small RNA categories in the CL and TL. [file 1471-2229-13-180-S4.doc]

**Table S4 Distribution of different small RNAs categories in CL and TL libraries**

| **Types of sequences** | **CL** | | | | **TL** | | | |
| --- | --- | --- | --- | --- | --- | --- | --- | --- |
|  | **Unique sRNAs** | **%** | **Total reads** | **%** | **Unique sRNAs** | **%** | **Total reads** | **%** |
| **total** | 3424945 | 100% | 12010658 | 100% | 3277192 | 100% | 12330771 | 100% |
| **rRNA** | 166812 | 4.87% | 2680356 | 22.32% | 165469 | 5.05% | 3717175 | 30.15% |
| **snRNA** | 1948 | 0.06% | 6175 | 0.05% | 2171 | 0.07% | 6990 | 0.06% |
| **snoRNA** | 989 | 0.03% | 2146 | 0.02% | 1061 | 0.03% | 2826 | 0.02% |
| **tRNA** | 24275 | 0.71% | 1071859 | 8.92% | 21381 | 0.65% | 1282932 | 10.40% |
| **exon_antisense** | 38416 | 1.12% | 84075 | 0.70% | 37151 | 1.13% | 77199 | 0.63% |
| **exon_sense** | 131349 | 3.84% | 212393 | 1.77% | 173217 | 5.29% | 255239 | 2.07% |
| **intron_antisense** | 34250 | 1.00% | 100126 | 0.83% | 32937 | 1.01% | 93174 | 0.76% |
| **intron_sense** | 39394 | 1.15% | 137867 | 1.15% | 38643 | 1.18% | 129974 | 1.05% |
| **unannotated** | 2939719 | 85.83% | 5811417 | 48.39% | 2758147 | 84.16% | 5262767 | 42.68% |
| **miRNA** | 47793 | 1.40% | 1904244 | 15.85% | 47015 | 1.43% | 1502495 | 12.18% |
