# Supplementary material for: Genome-wide identification of Thellungiella salsuginea microRNAs with putative roles in the salt stress response
Source: BMC Plant Biol. 2013 Nov 15;13:180. doi: 10.1186/1471-2229-13-180 (PMC4225614; doi:10.1186/1471-2229-13-180)
Supplement: Additional file 5: Table S5 — Detailed information of known miRNAs identified in Thellungiella salsuginea. [file 1471-2229-13-180-S5.rtf]

Table S6  Detail information of known miRNAs identified in Thellungiella salsuginea
miRNA 
family	MiRNA name	Length	Count 
CL	Count 
TL	Ratio 
(TL/CL)	sequence£¨5'-3'£©	miR*	Referenece
miRNAs	conserved miRNA homology
 in other plants	Pre-miRNAs location	
									 ath	ptc	vvi	osa	bna		
	conserved miRNAs	
miR156
/157	tsa-miR156a	20	665846	394884	0.609 	UGACAGAAGAGAGUGAGCAC	+	ath-miR156a	**	**	**	**	**	scaffold_15:4948287:4948425:-	
	tsa-miR156b	21	318	202	0.652 	CUGACAGAAGAUAGAGAGCAC		smo-miR156b	*	*	*	*	*		
	tsa-miR156b-3p	22	99	82	0.850 	GCUCACCUCUCUUUCUGUCAGU	+	aly-miR156b-3p	*	*	*	*	*	scaffold_1:3452779:3452903:+	
	tsa-miR156c	20	121	76	0.645 	UGUCAGAAGAGAGUGAGCAC		ghr-miR156c	*	*	*	*	*		
	tsa-miR156e	20	2082	1378	0.680 	UGACAGAAGAGAGCGAGCAC		zma-miR156k	*	*	*	*	*		
	tsa-miR156g	20	602	414	0.706 	CGACAGAAGAGAGUGAGCAC		ath-miR156g	**	*	*	*	*		
	tsa-miR156h	20	384	273	0.730 	UGACAGAAGAAAGAGAGCAC		ath-miR156h	**	*	*	*	*	scaffold_2:10225700:10225805:+	
	tsa-miR156i	21	7318	5274	0.740 	UGACAGAAGAGAGUGAGCACA	+	bna-miR156a	*	*	*	*	**	scaffold_1:3452779:3452903:+	
	tsa-miR156j	20	2732	1464	0.550 	UGACAGAAGAGAGAGAGCAC		ath-miR156j	**	*	*	**	*		
	tsa-miR156k	21	26	21	0.829 	UGACAGAAGAGAGAGAGCACA		osa-miR156k	*	*	*	**	*		
	tsa-miR156l	20	356	202	0.583 	UGACAGAAGAGGGUGAGCAC		mtr-miR156j	*	*	*	*	*		
	tsa-miR156m	20	306	182	0.611 	UGACAGAGGAGAGUGAGCAC		vvi-miR156e	*	*	**	*	*		
	tsa-miR156n	21	489	342	0.718 	UUGACAGAAGAAAGAGAGCAC		smo-miR156c	*	*	*	*	*	scaffold_2:10225700:10225805:+	
	tsa-miR156o	21	1825	736	0.414 	UUGACAGAAGAGAGAGAGCAC		ahy-miR156c	*	*	*	*	*		
	tsa-miR156p	20	1703	837	0.505 	UGACAGAAGAGAGGGAGCAC		ptc-miR156k	*	**	**	*	*		
	tsa-miR156q	21	66529	47753	0.737 	UUGACAGAAGAGAGUGAGCAC	+	gma-miR156k	*	*	*	*	*	scaffold_2:3514670:3514799:-	
	tsa-miR156r	21	386	236	0.628 	UUGACAGAAGAUAGAGGGCAC		mtr-miR156g	*	*	*	*	*		
	tsa-miR156s	22	25	16	0.657 	UUUGACAGAAGAUAGAGAGCAC	+	bgy-miR156	*	*	*	*	*	scaffold_13:3051829:3052046:+	
	tsa-miR157a	21	454526	314730	0.711 	UUGACAGAAGAUAGAGAGCAC	+	ath-miR157a	**	**	**	**	**	scaffold_9:9318600:9318731:+	
	tsa-miR157d	20	22402	15501	0.710 	UGACAGAAGAUAGAGAGCAC	+	ath-miR157d	**	**	**	**	**	scaffold_9:9304809:9304939:-	
miR158	tsa-miR158a	20	12	9	0.770 	UCCCAAAUGUAGACAAAGCA		ath-miR158a	**						
miR159	tsa-miR159a	21	2822	2053	0.747 	UUUGGAUUGAAGGGAGCUCUA	+	ath-miR159a	**	**	**	*	**	scaffold_9:3074886:3075085:+	
	tsa-miR159b	21	9	16	1.825 	UUUGGAUUGAAGGGAGCUCUU		ath-miR159b	**	*	*	*	*		
	tsa-miR160a	21	33	74	2.302 	UGCCUGGCUCCCUGUAUGCCA	+	ath-miR160a	**	**	**	**	**	scaffold_1:9864398:9864521:-	
	tsa-miR160a-3p	21	2809	2000	0.731 	GCGUAUGAGGAGCCAUGCAUA	+	ptc-miR160-3p	*	**	*	*	*	scaffold_10:10128504:10128624:+	
	tsa-miR160b-3p	21	56	103	1.888 	GCGUGCAAGGAGCCAAGCAUG	+	zma-miR160b-3p	*	*	*	**	*	scaffold_20:2068305:2068426:-	
miR161	tsa-miR161.2	21	104	88	0.869 	UCAAUGCAUUGAAAGUGACUA		ath-miR161.2	**				*	scaffold_7:5863828:5863942:-	
	tsa-miR161.1	21	7959	9945	1.283 	UUGAAAGUGACUACAUCGGGG		aly-miR161.1	*					scaffold_7:5863828:5863942:-	
miR162	tsa-miR162	20	40	37	0.950 	UCGAUAAACCUCUGCAUCCA	+	zma-miR162	*	*	*	*	*	scaffold_2:2641947:2642072:-	
	tsa-miR162-3p	22	35	66	1.936 	UGGAGGCAGCGGUUCAUCGAUC		csi-miR162-3p	*	*	*	*	*		
	tsa-miR162a	21	6461	4702	0.747 	UCGAUAAACCUCUGCAUCCAG	+	ath-miR162a	**	**	**	**	*	scaffold_2:2641947:2642072:-	
	tsa-miR162a-3p	21	28	27	0.990 	GGAGGCAGCGGUUCAUCGAUC	+	aly-miR162a-3p						scaffold_2:2641947:2642072:-	
miR164	tsa-miR164a	21	8745	9298	1.092 	UGGAGAAGCAGGGCACGUGCA		ath-miR164a	**	**	**	**	**	scaffold_22:1698100:1698421:+	
	tsa-miR164b	20	35	18	0.528 	UGGAGAAGCAGGGCACGUGC		gma-miR164b	*	*	*	*	*	scaffold_2:113814:113972:-	
	tsa-miR164c	21	763	811	1.091 	UGGAGAAGCAGGGCACGUGCG	+	ath-miR164c	**	*	*	*	**	scaffold_6:4116901:4117002:-	
	tsa-miR164d	21	165	155	0.964 	UGGAGAAGCAGGGCACGUGCU		osa-miR164d	*	*	*	**	*		
miR165/166	tsa-miR165a	21	940	1220	1.332 	UCGGACCAGGCUUCAUCCCCC	+	ath-miR165a	**	*	*	*	**	scaffold_5:15258725:15258867:-	
	tsa-miR166a	21	22306	27275	1.255 	UCGGACCAGGCUUCAUUCCCC	+	ath-miR166a	**	**	**	**	**	scaffold_14:2294318:2294494:+	
	tsa-miR166b-3p	21	36	29	0.827 	GGACUGUUGUCUGGCUCGAGG		aly-miR166b-3p				*			
	tsa-miR166c-3p	21	516	301	0.599 	GGAAUGUUGUCUGGCUCGAGG	+	zma-miR166c-3p				*		scaffold_22:1329544:1329726:+	
	tsa-miR166e	21	2	13	6.673 	UCGAACCAGGCUUCAUUCCCC		osa-miR166e	*	*	*	**	*		
	tsa-miR166e-3p	21	44	47	1.097 	GGAAUGUUGUCUGGCACGAGG	+	aly-miR166e-3p				*		scaffold_14:2294318:2294494:+	
	tsa-miR166f	19	435	679	1.603 	UCGGACCAGGCUUCAUUCC	+	vvi-miR166a	*	*	**	*	*	scaffold_14:2294318:2294494:+	
	tsa-miR166g	21	441	491	1.143 	UCGGACCAGGCUUCAUUCCUC	+	ptc-miR166n	*	**	*	**	*	scaffold_3:546347:546498:+	
	tsa-miR166h	20	362	506	1.435 	UCGGACCAGGCUUCAUUCCC	+	zma-miR166h	*	*	*	*	**	scaffold_22:1329544:1329726:+	
	tsa-miR166k	20	33	46	1.431 	UCGGACCAGGCUUCAUUCCU	+	sbi-miR166k	*	*	*	*	*	scaffold_3:546347:546498:+	
	tsa-miR166l	20	34	37	1.117 	UCGGACCAGGCUUCAUCCCC	+	aly-miR165a	*	*	*	*	*	scaffold_5:15258725:15258867:-	
	tsa-miR166m	21	34	23	0.695 	UCGGACCAGGCUUCAUUCCCU		osa-miR166m	*	*	*	**	*		
	tsa-miR166n	21	10	10	1.027 	UCGGACCAGGCUUCAUUCCUU		ptc-miR166n	*	**	*	*	*		
miR167	tsa-miR167a	21	82561	77109	0.959 	UGAAGCUGCCAGCAUGAUCUA	+	ath-miR167a	**	**	**	**	**	scaffold_17:630506:630624:+	
	tsa-miR167a-3p	21	51	28	0.564 	GAUCAUGUUCGCAGUUUCACC	+	aly-miR167a-3p				*		scaffold_13:868555:868695:-	
	tsa-miR167b	21	84	60	0.733 	UGAAGCUGACAGCAUGAUCUA		tae-miR167b	*	*	*	*	*		
	tsa-miR167c	21	133	107	0.826 	UAAGCUGCCAGCAUGAUCUUG	+	ath-miR167c	**	*	*	*	*	scaffold_13:8096158:8096344:+	
	tsa-miR167d	22	249	246	1.014 	UGAAGCUGCCAGCAUGAUCUGG		ath-miR167d	**	*	*	*	*		
	tsa-miR167e	22	115	119	1.062 	UGAAGCUGCCAGCAUGAUCUAA	+	bna-miR167a	*	*	*	*	**	scaffold_13:868555:868695:-	
	tsa-miR167f	21	660	632	0.983 	UGAAGCUGCCAGCAUGAUCUU		ptc-miR167f	*	**	*	*	*		
	tsa-miR167g	22	104	74	0.731 	UGAAGCUGCCAGCAUGAUCUGA		ccl-miR167a	*	*	*	*	*		
	tsa-miR167h	21	150	128	0.876 	UGAAGCUGCCAGCAUGAUCUC		vvi-miR167c	*	*	**	*	*		
	tsa-miR167i	21	68	0	0.000 	UGAAGCUGCCAGCAUGAUCUG		osa-miR167d	*	**	*	**	*		
miR168	tsa-miR168a	21	13089	19511	1.530 	UCGCUUGGUGCAGGUCGGGAA	+	ath-miR168a	**	**	**	*	**	scaffold_1:8872731:8872872:-	
	tsa-miR168d	20	19	43	2.323 	UCGCUUGGUGCAGGUCGGGA	+	cme-miR168	*	*	*	*	*	scaffold_20:1047212:1047352:-	
	tsa-miR168a-3p	21	319	215	0.692 	CCCGCCUUGCAUCAACUGAAU	+	aly-miR168a-3p	*	**	*	*	*	scaffold_1:8872731:8872872:-	
miR169	tsa-miR169a	21	161	143	0.912 	CAGCCAAGGAUGACUUGCCGA	+	ath-miR169a	**	**	**	**	**	scaffold_13:5033385:5033590:+	
	tsa-miR169b	21	209	105	0.516 	CAGCCAAGGAUGACUUGCCGG	+	ath-miR169b	**	**	**	**	**	scaffold_6:3362862:3363014:+	
	tsa-miR169c	21	1346	823	0.628 	UGAGCCAAAGAUGACUUGCCG	+	mtr-miR169i	*	*	*	*	**	scaffold_7:919581:919676:-	
	tsa-miR169d	21	43	35	0.836 	UGAGCCAAGGAUGACUUGCCG	+	ath-miR169d	**	**	*	*	*	scaffold_1:7989377:7989480:+	
	tsa-miR169e	20	10	0	0.000 	AGCCAAGGAUGACUUGCCGG		gma-miR169e	*	*	*	*	*	scaffold_14:4172294:4172517:+	
	tsa-miR169b-3p	22	50	29	0.595 	GGCAAGUUGUCCUUCGGCUACA	+	aly-miR169b-3p	*	*	*	*	*	scaffold_6:3362862:3363014:+	
miR171	tsa-miR171a	21	60	74	1.266 	UGAUUGAGCCGCGCCAAUAUC	+	ath-miR171a	**	*	**	**	**	scaffold_16:1985605:1985730:-	
	tsa-miR171c	21	12	14	1.198 	UGAUUGAGCCGUGCCAAUAUC		osa-miR171b	*	**	**	**	*	scaffold_16:3464453:3464559:-	
	tsa-miR171d	20	10	12	1.232 	UUGAGCCGUGCCAAUAUCAC	+	zma-miR171b	*	*	*	*	*	scaffold_5:11619062:11619184:+	
	tsa-miR171f	21	198	112	0.581 	AGAUAUUAGUGCGGUUCAAUC	+	aly-miR171b	*	*	*	*	*	scaffold_5:11619062:11619184:+	
	tsa-miR171g	21	240	164	0.702 	AGAUAUUGGUGCGGUUCAAUC	+	aly-miR171c	*	*	*	*	*	scaffold_8:2266346:2266469:+	
miR172	tsa-miR172a	21	14702	14124	0.986 	AGAAUCUUGAUGAUGCUGCAU	+	ath-miR172a	**	**	**	**	**	scaffold_10:3236505:3236622:-	
	tsa-miR172b	21	31	24	0.795 	GCAGCACCAUCAAGAUUCACA	+	aly-miR172b	*	*	*	*	*	scaffold_2:1159895:1160021:-	
	tsa-miR172c	21	114	139	1.252 	AGAAUCUUGAUGAUGCUGCAG	+	ath-miR172c	**	*	*	*	**	scaffold_16:222566:222679:-	
	tsa-miR172d	23	32	30	0.962 	UGAGAAUCUUGAUGAUGCUGCAU	+	vvi-miR172d	*	*	**	*	*	scaffold_2:1159895:1160021:-	
	tsa-miR172e	21	174	149	0.879 	GGAAUCUUGAUGAUGCUGCAU	+	ath-miR172e	**	**	*	**	**	scaffold_2:8433221:8433362:-	
	tsa-miR172f	20	314	179	0.585 	GAAUCUUGAUGAUGCUGCAU	+	aly-miR172e	*	*	*	*	*	scaffold_2:8433221:8433362:-	
	tsa-miR172h	21	7	12	1.760 	AGAAUCCUGAUGAUGCUGCAU		tcc-miR172d	*	*	*	*	*		
	tsa-miR172i	20	46	50	1.116 	AGAAUCUUGAUGAUGCUGCA	+	zma-miR172a	*	*	*	*	*	scaffold_16:222566:222679:-	
miR319	tsa-miR319a	21	4	3	0.770 	UUGGACUGAAGGGAGCUCCCU		ath-miR319a	**	*	**	*	*	scaffold_3:4841417:4841605:-	
	tsa-miR319c	21	4	0	0.000 	UUGGACUGAAGGGAGCUCCUU		ath-miR319c	**	*	*	*	*		
miR390	tsa-miR390a	21	1107	854	0.792 	AAGCUCAGGAGGGAUAGCGCC	+	ath-miR390a	**	**	**	**	**	scaffold_10:9718512:9718623:+	
miR391	tsa-miR391	21	4	7	1.797 	UUCGCAGGAGAGAUAGCGCCA		ath-miR391	**						
miR393	tsa-miR393a	21	1	1	1.027 	UCCAAAGGGAUCGCAUUGAUC	+	osa-miR393	**	**	**	**	**	scaffold_16:74495:74651:-	
	tsa-miR393b-3p	21	54	28	0.532 	AUCAUGCGAUCUCUUUGGAUU	+	aly-miR393b-3p						scaffold_16:74495:74651:-	
miR394	tsa-miR394a	20	16	30	1.925 	UUGGCAUUCUGUCCACCUCC		ath-miR394a	**	**	**	**	**		
miR395	tsa-miR395a	21	31	35	1.159 	CUGAAGUGUUUGGGGGAACUC		ath-miR395a	**	**	**	*	**	scaffold_5:6676074:6676166:-	
	tsa-miR395b	21	95	135	1.459 	CUGAAGUGUUUGGGGGGACUC		ath-miR395b	**	*	*	*	**	scaffold_5:6677672:6677777:+	
miR396	tsa-miR396a	21	171	168	1.009 	UUCCACAGCUUUCUUGAACUG	+	ath-miR396a	**	**	**	**	**	scaffold_11:11322339:11322491:+	
	tsa-miR396b	21	66	50	0.778 	UUCCACAGCUUUCUUGAACUU	+	ath-miR396b	**	**	*	**	**	scaffold_18:2696064:2696206:+	
	tsa-miR396a-3p	21	117	48	0.421 	GUUCAAUAAAGCUGUGGGAAG	+	aly-miR396a-3p				*		scaffold_11:11322339:11322491:+	
	tsa-miR396b-3p	21	432	275	0.654 	GCUCAAGAAAGCUGUGGGAAA	+	aly-miR396b-3p				*		scaffold_18:2696064:2696206:+	
miR398	tsa-miR398a	21	1	5	5.133 	UGUGUUCUCAGGUCACCCCUG	+	ath-miR398b	**	*	*	*		scaffold_2:4759551:4759663:+	
miR399	tsa-miR399a	21	9	4	0.456 	UGCCAAAGGAGAGUUGCCCUG	+	ath-miR399b	**	*	**	**	*	scaffold_6:2179564:2179686:-	
miR408	tsa-miR408	21	192	391	2.091 	AUGCACUGCCUCUUCCCUGGC	+	ath-miR408	**	**	**	*	*	scaffold_22:1478349:1478457:+	
	tsa-miR408-5p	21	972	4653	4.915 	CAGGGAACAAGCAGAGCAUGG	+	aly-miR408-5p		*		*		scaffold_22:1478349:1478457:+	
	non-conserved miRNAs	
miR2111a	tsa-miR2111a	21	10	4	0.411 	UAAUCUGCAUCCUGAGGUUUA	+	ath-miR2111a	**	*	*		**	scaffold_13:6545861:6546008:-	
	tsa-miR2111b-3p	21	25	22	0.903 	AUCCUCGGGAUACAGAUUACC	+	bna-miR2111b*	*		*		**	scaffold_2:428924:429045:+	
miR400	tsa-miR400	21	316	215	0.699 	UAUGAGAGUAUUAUAAGUCAC	+	ath-miR400	**					scaffold_5:2382635:2382736:-	
miR403	tsa-miR403	21	632	474	0.770 	UUAGAUUCACGCACAAACUCG	+	ath-miR403	**	**	**		**	scaffold_22:1579827:1579948:+	
miR824	tsa-miR824	21	6468	4585	0.728 	UAGACCAUUUGUGAGAAGGGA		ath-miR824	**				**		
	tsa-miR824-3p	21	109	82	0.772 	CCUUCUCAUCGAUGGUCUAGA		aly-miR824*							
miR827	tsa-miR827b	21	1428	1238	0.890 	UUAGAUGACCAUCAACAAACG	+	aly-miR827	*	*		*		scaffold_19:1282364:1282476:+	
miR845	tsa-miR845a	21	98	91	0.953 	CGGCUCUGAUACCAAUUGAUG		ath-miR845a	**		*			scaffold_1:7177132:7177214:+	
miR5139	tsa-miR5139	19	14	25	1.833 	AAACCUGGCUCUGAUACCA		rgl-miR5139							
miR4995	tsa-miR4995	21	4	11	2.823 	AGGCAGUGGCUUGGUUAAGGG		gma-miR4995							
miR894	tsa-miR894	20	42	209	5.109 	CGUUUCACGUCGGGUUCACC		ppt-miR894		**					


The abbreviations represent: ath, Arabidopsis thaliana; ptc, Populus trichocarpa; vvi, Vitis vinifera; osa, Oryza sativa; bna, Brassica napus. The plus symbols indicate: **, miRNA sequences of Thellungiella were exactly identical to those in other species; *, miRNA sequences of Thellungiella were conserved in other species but have variations in some nucleotide positions. The # means more than one pre-miRNA locations in the genome. The + in the column of star means the star* sequences were identified.
