# Supplementary material for: Genome-wide identification of Thellungiella salsuginea microRNAs with putative roles in the salt stress response
Source: BMC Plant Biol. 2013 Nov 15;13:180. doi: 10.1186/1471-2229-13-180 (PMC4225614; doi:10.1186/1471-2229-13-180)
Supplement: Additional file 6: Figure S1 — Hairpin structures of known miRNAs of Thellungiella. [file 1471-2229-13-180-S6.rtf]

(1) tsa-miR156a: UGACAGAAGAGAGUGAGCAC

A     AAA---   AAA   A       -     -         A   ---|     UU    UAUGU-   U 
 AAAGA      CAC   GAA CUGACAG AAGAG AGUGAGCAC CAA   AGGCAA  UGCA      UUG A
 UUUCU      GUG   CUU GACUGUC UUCUC UCACUCGUG GUU   UUCGUU  ACGU      AGC U
G     AUAGUC   GC-   A       U     G         C   CUC^     U-    CACUCU   C

(2) tsa-miR156b-3p: GCUCACCUCUCUUUCUGUCAGU
 
GAAA  C   C     A------         -      -|       AU   G   CU  U   U 
    UG AAA AGAGA       AACUGACAG AAGAGA GUGAGCAC  GCA GCA  GU AUG G
    AC UUU UCUCU       UUGACUGUC UUCUCU CACUCGUG  UGU CGU  CA UAC U
C---  -   C     CCGUCCG         U      C^       CG   G   UU  U   C

(3) tsa-miR156h: UGACAGAAGAAAGAGAGCAC

A   AAA   U  A      -            A-|    GAUU       AAG 
 UGA   AUG UG CAGAAG AAAGAGAGCACA  CCCGG    AGCAAAA   \
 GCU   UAC AC GUCUUC UUUCUCUCGUGU  GGGCU    UCGUUUU   A
C   AC-   C  C      A            GA^    GUUC       GAU

(4) tsa-miR156i: UGACAGAAGAGAGUGAGCACA

-      AAAC--  AUC   A        -    -         A   ---|    CUU     GU 
 UAGAGA      GC   GAA CUGACAGA AGAG AGUGAGCAC CAA   AGGCA   UGCAU  \
 AUUUCU      CG   CUU GACUGUCU UCUC UCACUCGUG GUU   UUCGU   ACGUA  U
U      GAGCCA  GC-   A        A    G         C   CUC^    UU-     GC

(5) tsa-miR156n: UUGACAGAAGAAAGAGAGCAC

A   AAA   U  A      -            A-|    GAUU       AAG 
 UGA   AUG UG CAGAAG AAAGAGAGCACA  CCCGG    AGCAAAA   \
 GCU   UAC AC GUCUUC UUUCUCUCGUGU  GGGCU    UCGUUUU   A
C   AC-   C  C      A            GA^    GUUC       GAU

(6) tsa-miR156q: UUGACAGAAGAGAGUGAGCAC

-----|  G        AAA     --        -            A      G  AG     UAAAAA 
     GUU AUGGGGGA   GAAGU  UGACAGAA GAGAGUGAGCAC CAAAGG GA  UUGUA      \
     UAA UACUUCCU   CUUCA  ACUGUCUU CUCUCACUCGUG GUUUUC UU  GGCAU      U
AUUUC^  G        ---     AU        U            C      G  --     UUUCUC

(7) tsa-miR156s: UUUGACAGAAGAUAGAGAGCAC 

UUU---      G   UU   U U         -           .-UAA     .-AC|   A 
      GAGAAU AUG  GGU G UGACAGAAG AUAGAGAGCAC     GGAUG    AUGC A
      UUCUUA UAU  CCA C ACUGUCUUC UAUCUCUCGUG     CUUAU    UACG G
UUCUCC      -   UU   C -         A           \ ---     \ --^   U 
                                       

(8) tsa-miR157a: UUGACAGAAGAUAGAGAGCAC

UUUGG      U-    U-   U         -              U  -| A       UUC  AG 
     GAGGCG  UGAU  GUG UGACAGAAG AUAGAGAGCACAGA GA UG GAUGCAA   AG  \
     UUCCGU  AUUA  UAC ACUGUCUUC UAUCUCUCGUGUUU CU AU CUACGUU   UC  C
CCAA-      UU    UC   U         G              C  C^ A       UCA  GA

(9) tsa-miR157d: UGACAGAAGAUAGAGAGCAC

UUUGAG     U-   U-    U         A-             U  -|     C   UUCGGA 
      AGGCA  UGA  AGUG UGACAGAAG  UAGAGAGCACAGA GA UGAGAU CAA      A
      UUUGU  AUU  UCAC ACUGUCUUC  AUUUCUCGUGUUU CU AUUCUA GUU      C
CCUAA-     UU   UU    U         CG             C  C^     C   UUUUGA

(10) tsa-miR159a:UUUGGAUUGAAGGGAGCUCUA

A    U   A            AA        C      UU-   ------|      AGA      G  A  C    GAUC     AGC        GUAUA 
 AAGA GGA GUAGAGCUCCUU  AGUUCAAA GAGAGU   AGU      AGGGUAA   AAAGCU CU AG UAUG    CCAUA   CCUAAUCC     A
 UUCU UCU CAUCUCGAGGGA  UUAGGUUU UUCUCA   UCG      UCCCGUU   UUUCGA GA UC AUAC    GGUAU   GGAUUAGG     A
C    -   A            AG        C      UUU   GUAAUU^      CAA      G  C  U    GUUC     CUU        AAAAG

(11) tsa-miR160a: UGCCUGGCUCCCUGUAUGCCA

UAUAUAUGG----|   AUA       C     CC-           A   AAGCUC      CAU 
             GUUU   AUCGUGC UGGCU   CUGUAUGCCAC AGU      CGAUUU   \
             CGAA   UAGUACG ACUGA   GACAUACGGUG UCA      GCUAAA   A
AUAUAGUUCUUUA^   AC-       A     UGA           A   CUA---      ACA

(12) tsa-miR160a-3p: GCGUAUGAGGAGCCAUGCAUA

A        U     A  ----|    C       CU         A  AU   C      AG 
 UAUAUAUA UGUAU UG    UAUGC UGGCUCC  GUAUGCCAU CA  GAG UCAUCG  \
 AUAUAUAU ACAUA AC    AUACG ACCGAGG  UAUGCGGUA GU  UUC AGUAGC  U
-        -     C  UCCU^    U       AG         G  GC   C      UA

(13) tsa-miR160b-3p: GCGUGCAAGGAGCCAAGCAUG

-   AUA   AUUCACC-|      C       C          GA UG AUA        AG 
 UAU   UUG        GUCAUGC UGGCUCC UGUAUGCCAC  G  G   CUGGUUUU  \
 AUG   AAC        CAGUACG ACCGAGG ACGUGCGGUG  C  C   GGCUAAAA  C
A   GUA   GAAAUUAC^      A       A          GC GU ---        GU

(14) tsa-miR161.2: UCAAUGCAUUGAAAGUGACUA

-|  C      U         C       G    UGU     G     C    C  UG 
 GAU AAUGCA UGAAAGUGA UACAUCG GGCU   UUUUU UUUUU UCAG UU  \
 CUA UUGCGU ACUUUCACU AUGUGGC CCGA   AAAAG AGAAG AGUU AA  A
A^  A      C         A       A    UU-     G     U    U  CU

(15) tsa-miR161.1: UUGAAAGUGACUACAUCGGGG

-|  C      U         C       G    UGU     G     C    C  UG 
 GAU AAUGCA UGAAAGUGA UACAUCG GGCU   UUUUU UUUUU UCAG UU  \
 CUA UUGCGU ACUUUCACU AUGUGGC CCGA   AAAAG AGAAG AGUU AA  A
A^  A      C         A       A    UU-     G     U    U  CU

(16) tsa-miR162: UCGAUAAACCUCUGCAUCCA 

A|  UGA   U       G    C    C        C   C  GGAU      ----      UG U 
 GAG   AAG CGCUGGA GCAG GGUU AUCGAUCU UUC UG    UUUUUG    UUUUUU  G \
 CUC   UUU GCGACCU CGUC CCAA UAGCUAGA AAG AC    GAAAAC    AGAAAG  C U
-^  CG-   -       A    U    A        C   U  ----      UUAA      GU G

(17) tsa-miR162a: UCGAUAAACCUCUGCAUCCAG
tsa-miR162a-3p: GGAGGCAGCGGUUCAUCGAUC

A|  UGA   U       G    C    C        C   C  GGAU      ----      UG U 
 GAG   AAG CGCUGGA GCAG GGUU AUCGAUCU UUC UG    UUUUUG    UUUUUU  G \
 CUC   UUU GCGACCU CGUC CCAA UAGCUAGA AAG AC    GAAAAC    AGAAAG  C U
-^  CG-   -       A    U    A        C   U  ----      UUAA      GU G


(18) tsa-miR164a: UGGAGAAGCAGGGCACGUGCA 

GACAAG  A    U  GA   A       U    CCUCUUCA  C       .-UUUCUUCUUCAACCCUUCUUUU       .-A|   AU 
      GC UUGU UG  CCU GUUUCUC UAGC        CU UCUCACU                        GCGUGAG   UGCC  \
      CG AACG GC  GGG CGAAGAG GUUG        GA AGAGUGG                        CGCACUC   ACGG  C
AAAUAA  A    U  AC   A       -    CACCUC--  -       \ ----------------------       \ -^   UA 


(19) tsa-miR164b: UGGAGAAGCAGGGCACGUGC

C| AG  A     A    CA         CAUU         UCUCUUUGCAUAUA   A   G   U    CUAA      G  UG 
 UG  CA GAUGG GAAG  GGGCACGUG    ACUAGCUCA              UAC CAU CAC CUCC    AUGCAU UA  \
 AC  GU CUACC CUUC  CCCGUGUAC    UGAUUGAGU              AUG GUA GUG GAGG    UGUGUA AU  A	
C^ CA  A     C    UA         UUCU         UA------------   -   -   U    UG--      G  UU

(20) tsa-miR164c: UGGAGAAGCAGGGCACGUGCG

UA|   U  C     A  C             GCAC     A    UAU  GU 
  ACAC UG UGGAG AG AGGGCACGUGCGA    AAAUG GAUC   CG  \
  UGUG AC ACCUC UC UCUUGUGCACGCU    UUUAU CUAG   GU  A
UG^   C  A     C  A             ----     A    UU-  AC


(21) tsa-miR165a: UCGGACCAGGCUUCAUCCCCC

UUAA-  U  UU  -    A    A   UU      A           .-A|       GCC 
     GC AU  CA GUUG GGGG AUG  GUCUGG UCGAGGAUAUU   UAUAUAUA   A
     CG UA  GU CAAC CCCC UAC  CGGACC GGCUCCUAUGA   AUAUAUGU   U
GUCUC  U  UU  A    C    C   UU      A           \ -^       ACA


(22) tsa-miR166a: UCGGACCAGGCUUCAUUCCCC

----|    UUUUUCCUU            UU      CA   GGCCC     U  GAUCUAC          A   A      UCGCUGCUC 
    GAAGC         UUGAGGGGAAUG  GUCUGG  CGA     UUAAC CA       AAUUGAUUAU UAU UAUAUA         \
    CUUCG         AACUCCCCUUAC  CGGACC  GCU     AAUUG GU       UUAGCUAGUA GUA AUAUAU         A
CUAG^    UUACAUU--            UU      AG   GCAGU     -  AUAU---          A   C      UUACCAAUA


(23) tsa-miR166c-3p: GGAAUGUUGUCUGGCUCGAGG

----    -  UUUCUCUU   A        UU      CU   G   U    CUUU     .-U|  U        UU 
    AGGG GC        UUG GGGGAAUG  GUCUGG  CGA GAC CUGG    CUCUA   UCA GUUGGAUC  C
    UCCC CG        AAC CCCCUUAC  CGGACC  GCU CUG GAUU    GGGAU   AGU UAAUCUAG  U
UUAG    U  UUAUU---   C        UU      AG   G   C    UU--     \ -^  -        CU 


(24) tsa-miR166e-3p: GGAAUGUUGUCUGGCACGAGG

----|    UUUUUCCUU            UU      CA   GGCCC     U  GAUCUAC          A   A      UCGCUGCUC 
    GAAGC         UUGAGGGGAAUG  GUCUGG  CGA     UUAAC CA       AAUUGAUUAU UAU UAUAUA         \
    CUUCG         AACUCCCCUUAC  CGGACC  GCU     AAUUG GU       UUAGCUAGUA GUA AUAUAU         A
CUAG^    UUACAUU--            UU      AG   GCAGU     -  AUAU---          A   C      UUACCAAUA

(25) tsa-miR166f: UCGGACCAGGCUUCAUUCC

----|    UUUUUCCUU            UU      CA   GGCCC     U  GAUCUAC          A   A      UCGCUGCUC 
    GAAGC         UUGAGGGGAAUG  GUCUGG  CGA     UUAAC CA       AAUUGAUUAU UAU UAUAUA         \
    CUUCG         AACUCCCCUUAC  CGGACC  GCU     AAUUG GU       UUAGCUAGUA GUA AUAUAU         A
CUAG^    UUACAUU--            UU      AG   GCAGU     -  AUAU---          A   C      UUACCAAUA

(26) tsa-miR166g: UCGGACCAGGCUUCAUUCCUC

A-     UAUUUCC-             UU      A    G   A    C-      A      ---|        A 
  GAGGC        GUUGAGGGGAAUG  GUUUGG UCGA GAU UCAU  AUAUGC UACGUA   UAAUAUCGU G
  UUCCG        CAACUCUCCUUAC  CGGACC GGCU CUA GGUA  UAUAUG AUGUAU   AUUAUAGCG A
AA     UUAUUCUA             UU      A    G   A    UC      G      AAC^        U

(27) tsa-miR166h: UCGGACCAGGCUUCAUUCCC

----    -  UUUCUCUU   A        UU      CU   G   U    CUUU     .-U|  U        UU 
    AGGG GC        UUG GGGGAAUG  GUCUGG  CGA GAC CUGG    CUCUA   UCA GUUGGAUC  C
    UCCC CG        AAC CCCCUUAC  CGGACC  GCU CUG GAUU    GGGAU   AGU UAAUCUAG  U
UUAG    U  UUAUU---   C        UU      AG   G   C    UU--     \ -^  -        CU 

(28) tsa-miR166k: UCGGACCAGGCUUCAUUCCU

A-     UAUUUCC-             UU      A    G   A    C-      A      ---|        A 
  GAGGC        GUUGAGGGGAAUG  GUUUGG UCGA GAU UCAU  AUAUGC UACGUA   UAAUAUCGU G
  UUCCG        CAACUCUCCUUAC  CGGACC GGCU CUA GGUA  UAUAUG AUGUAU   AUUAUAGCG A
AA     UUAUUCUA             UU      A    G   A    UC      G      AAC^        U

(29) tsa-miR166l: UCGGACCAGGCUUCAUCCCC

UUAA-  U  UU  -    A    A   UU      A           .-A|       GCC 
     GC AU  CA GUUG GGGG AUG  GUCUGG UCGAGGAUAUU   UAUAUAUA   A
     CG UA  GU CAAC CCCC UAC  CGGACC GGCUCCUAUGA   AUAUAUGU   U
GUCUC  U  UU  A    C    C   UU      A           \ -^       ACA

(30) tsa-miR167a: UGAAGCUGCCAGCAUGAUCUA

CACG    -    G---      A           G        AAA       C-  ---| GC 
    GUGC ACCA    CAUCUG UGAAGCUGCCA CAUGAUCU   CUUCUCU  UC   UC  U
    UACG UGGU    GUGGAC ACUUUGAUGGU GUACUAGA   GAAGGGA  AG   AG  C
----    C    AUUA      C           -        CUA       CU  UUU^ AU


(31) tsa-miR167a-3p: GAUCAUGUUCGCAGUUUCACC

UAUA-    AC   -  UCUGA         C            AUA      UU-    C-----|     UU 
     GUGC  CGG CA     UGAAGCUGC AGCAUGAUCUAA   ACUUUC   UCUC      GUUGUU  \
     CACG  GUC GU     ACUUUGACG UUGUACUAGAUU   UGAGAG   AGAG      UAGCAG  C
AGUCC    CU   A  UGCCC         C            AAA      UAC    AAAAGU^     UC

(32) tsa-miR167c: UAAGCUGCCAGCAUGAUCUUG

UAC     -  UAG---      UU-                  U    UC       .-AAGAUUCAU|UA 
   CGUGC AC      UAGCAG   AAGCUGCCAGCAUGAUCU GUCU  CUCUCUC           G  \
   GUACG UG      AUCGUC   UUUGAUGGUCGUACUGGA CAGA  GAGAGAG           C  U
A--     A  UUAGUA      CAC                  U    GA       \ ---------^AA 


(33) tsa-miR167e: UGAAGCUGCCAGCAUGAUCUAA

UAUA-    AC   -  UCUGA         C            AUA      UU-    C-----|     UU 
     GUGC  CGG CA     UGAAGCUGC AGCAUGAUCUAA   ACUUUC   UCUC      GUUGUU  \
     CACG  GUC GU     ACUUUGACG UUGUACUAGAUU   UGAGAG   AGAG      UAGCAG  C
AGUCC    CU   A  UGCCC         C            AAA      UAC    AAAAGU^     UC

(34) tsa-miR168a: UCGCUUGGUGCAGGUCGGGAA
tsa-miR168a-3p: CCCGCCUUGCAUCAACUGAAU

AG-      U  -     G      C          U     A      --|   G   C     CGU   UU 
   UCACCG CG GGCUC GAUUCG UUGGUGCAGG CGGGA CUAAUU  UGCU ACA GGCCA   GAG  \
   AGUGGC GC CCGAG CUAAGU AACUACGUUC GCCCU GGUUAG  ACGA UGU UUGGU   UUC  U
UAA      C  U     G      C          C     A      GG^   G   -     UGU   CU

(35) tsa-miR168d: UCGCUUGGUGCAGGUCGGGA

AG---     GG  GU   G      C          U     A      G--   G   CC   --|     C 
     UUACC  CG  CUC GAUUCG UUGGUGCAGG CGGGA CUGAUU   GCU ACA  GCC  ACGUGG U
     AGUGG  GC  GAG CUAAGU AACUACGUUC GCCCU GGCUAG   CGA UGU  CGG  UGUACC U
AGAAA     A-  UC   G      C          C     A      GGA   G   U-   UU^     G


(36) tsa-miR169a: CAGCCAAGGAUGACUUGCCGA

-   U      C         UG           UAAAUUU        AUUCUC---    AAAACAUU-  C  A     A     .-CCUG|      AAG 
 AAG AGUGUG AGCCAAGGA  ACUUGCCGAUU       AUAUUUUU         UCUU         UU UG UUUUC AAUGC      GUUCUUG   \
 UUC UUAUAC UCGGUUCCU  UGAACGGCUAG       UAUAAAAA         AGAA         AA AC AAAAG UUAUG      CAAGAAU   A
U   U      A         GU           -------        GAAAAACAA    ACAUUAAAU  A  -     A     \ ----^      GGA 


(37) tsa-miR169b: CAGCCAAGGAUGACUUGCCGG

A--   U   U      U    C    -     UG           -------     A   UGU     --|    A  GA 
   AGU GAG AGAGUA AAUG AGCC AAGGA  ACUUGCCGGAA       CGUUA CGU   UCAUC  CAUAU AA  U
   UCA CUU UCUCGU UUAC UCGG UUCCU  UGAACGGUCUU       GUAGU GUA   AGUAG  GUAUA UU  A
UAC   U   C      U    A    C     GU           UAUACGA     G   UU-     AA^    G  AU

(38) tsa-miR169c: UGAGCCAAAGAUGACUUGCCG

A|  U     U         A          AAUA    AAC     UC 
 GAA GAGAU GAGCCAAAG UGACUUGCCG    GACG   GAAUC  A
 CUU CUUUG CUCGGUUUC GUUGAACGGC    CUGU   UUUAG  A
A^  C     U         A          ----    GGU     UG

(39) tsa-miR169d: UGAGCCAAGGAUGACUUGCCG

-|   U    UU         A           UUUCUU      UG--    UA 
 AGAA GAGG  GAGCCAAGG UGACUUGCCGA      UACCAA    AAUC  A
 UCUU CUUU  CUCGGUUCC GUUGAACGGCU      GUGGUU    UUAG  A
C^   C    GU         A           U-----      UUAA    UA

(40) tsa-miR169e: AGCCAAGGAUGACUUGCCGG

AUU     U      AG           -   A-       .-AUAU|           CAU 
   GUUGU UAGCCA  GAUGACUUGCC GGC  GCUUUGU      AGCAUAAUUUUU   \
   CAACG AUCGGU  CUACUGAACGG CCG  CGAAACA      UCGUAUUAGAGG   C
CGU     U      CU           C   AA       \ ----^           UAU 

(41) tsa-miR169b-3p: GGCAAGUUGUCCUUCGGCUACA

A--   U   U      U    C    -     UG           -------     A   UGU     --|    A  GA 
   AGU GAG AGAGUA AAUG AGCC AAGGA  ACUUGCCGGAA       CGUUA CGU   UCAUC  CAUAU AA  U
   UCA CUU UCUCGU UUAC UCGG UUCCU  UGAACGGUCUU       GUAGU GUA   AGUAG  GUAUA UU  A
UAC   U   C      U    A    C     GU           UAUACGA     G   UU-     AA^    G  AU

(42) tsa-miR171a: UGAUUGAGCCGCGCCAAUAUC

GC---|   A     CC  UU         C       U      CU   U  CCACACAC   CAU 
     ACGA AGAGU  CU  GAUAUUGGC UGGUUCA UCAGAU  UCU GA        GCA   \
     UGCU UCUCG  GA  CUAUAACCG GCCGAGU AGUCUA  AGA CU        CGU   A
CUCUC^   C     U-  CC         C       U      U-   U  CUUA----   UAU 

(43) tsa-miR171c: UGAUUGAGCCGUGCCAAUAUC

A|    AA     C               G          AAUC    UAUCU 
 UUUCU  AGUAG GGUAUUGGUACGGUU AAUCGGAUCA    ACUC     U
 AAAGA  UUAUC CUAUAACCGUGCCGA UUAGUUUAGU    UGAG     U
-^    CA     U               G          AAAC    UUAAC

(44) tsa-miR171d: UUGAGCCGUGCCAAUAUCAC

UUUUGA   AA----   --  C   A      A UG          A     --|   G    UAG 
      UGU      GGU  AA GCG GAUAUU G  CGGUUCAAUC AAUAG  CCGA CUCU   U
      ACA      CCG  UU CGC CUAUAA C  GCCGAGUUAG UUGUC  GGCU GAGA   U
A-----   GUAGAG   AA  A   A      C GU          C     UU^   A    UAU

(45) tsa-miR171f: AGAUAUUAGUGCGGUUCAAUC

UUUUGA   AA----   --  C   A      A UG          A     --|   G    UAG 
      UGU      GGU  AA GCG GAUAUU G  CGGUUCAAUC AAUAG  CCGA CUCU   U
      ACA      CCG  UU CGC CUAUAA C  GCCGAGUUAG UUGUC  GGCU GAGA   U
A-----   GUAGAG   AA  A   A      C GU          C     UU^   A    UAU

(46) tsa-miR171g: AGAUAUUGGUGCGGUUCAAUC

U    .-AUA  A        UG          CAA-|     CA   CCUUU 
 GGCA     CG GAUAUUGG  CGGUUCAAUC    AAACCG  CUC     \
 UCGU     GC CUAUAACC  GCCGAGUUAG    UUUGGC  GAG     U
G    \ ---  A        GU          UUUG^     UA   AUGUU 


       C-----------    A 
                   GUUU A
                   CAAA U
       CGAUUCUACUUC    A 
    
(47) tsa-miR172a: AGAAUCUUGAUGAUGCUGCAU

A   U      C  UG               A     GUUUAU-|   GG   U  UUUA 
 UUG UUGCUG UG  GCAUCAUCAAGAUUC CAUCU       GGAC  UGG GA    \
 AAC AACGGC AC  CGUAGUAGUUCUAAG GUAGA       CUUG  ACC CU    C
-   U      U  GU               A     AGUGUCU^   AA   -  CUCU 

(48) tsa-miR172b: GCAGCACCAUCAAGAUUCACA

AAC--     U  U    A         C           ------     -  UC-|       UUA 
     AGUCG UG UUGU GGUGCAGCA CAUCAAGAUUC      ACAUA CA   UCCUAAUU   \
     UCGGC GC AACA CUACGUCGU GUAGUUCUAAG      UGUAU GU   GGGAUUAA   U
AAACA     C  C    G         A           AGUAUA     A  UUU^       CUA

(49) tsa-miR172c: AGAAUCUUGAUGAUGCUGCAG

UUGU|   UA     U    U         AG        UAUUUU         UCU 
    UUGC  UUGCA CAUC UCAAGAUUC  AAAUCAGA      GAUGGGUUC   \
    AACG  GACGU GUAG AGUUCUAAG  UUUGGUUU      CUAUCCGAG   U
AAUU^   GC     C    U         AG        CUU---         UUU 

(50) tsa-miR172d: UGAGAAUCUUGAUGAUGCUGCAU

AAC--     U  U    A         C           ------     -  UC-|       UUA 
     AGUCG UG UUGU GGUGCAGCA CAUCAAGAUUC      ACAUA CA   UCCUAAUU   \
     UCGGC GC AACA CUACGUCGU GUAGUUCUAAG      UGUAU GU   GGGAUUAA   U
AAACA     C  C    G         A           AGUAUA     A  UUU^       CUA

(51) tsa-miR172e: GGAAUCUUGAUGAUGCUGCAU

AAC-     -|  G    A                     A  AG   UGU  UU      G      CCUC 
    AGCCG GUA UUGC GAUGCAGUAUCAUUAAGAUUC CA  CGA   GG  UCCUUU UUUUCG    U
    UCGGU CAU AACG CUACGUCGUAGUAGUUCUAAG GU  GCU   CC  GGGAAA AAAAGU    C
AGUC     A^  A    A                     G  AA   UU-  UU      -      CUGA

(52) tsa-miR172f: GAAUCUUGAUGAUGCUGCAU

AAC-     -|  G    A                     A  AG   UGU  UU      G      CCUC 
    AGCCG GUA UUGC GAUGCAGUAUCAUUAAGAUUC CA  CGA   GG  UCCUUU UUUUCG    U
    UCGGU CAU AACG CUACGUCGUAGUAGUUCUAAG GU  GCU   CC  GGGAAA AAAAGU    C
AGUC     A^  A    A                     G  AA   UU-  UU      -      CUGA

(53) tsa-miR172i: AGAAUCUUGAUGAUGCUGCA

UUGU|   UA     U    U         AG        UAUUUU         UCU 
    UUGC  UUGCA CAUC UCAAGAUUC  AAAUCAGA      GAUGGGUUC   \
    AACG  GACGU GUAG AGUUCUAAG  UUUGGUUU      CUAUCCGAG   U
AAUU^   GC     C    U         AG        CUU---         UUU

(54) tsa-miR319a: UUGGACUGAAGGGAGCUCCCU

AAUAACAAAA|         A      UUCACAU        AU  AU--    C  CC     -   AC      A        C     --    AAA 
          GAGCUUCCUU AGUCCA       GUUGUAAU  AU    GAUC AA  AGUUU CGG  UCAUUC UUCAUUUA CAAGU  AGUU   \
          CUCGAGGGAA UCAGGU       UAGCGUUA  UG    CUAG UU  UCAGA GCU  AGUAAG GAGUAAAU GUUCA  UCAG   A
UUUUUUUUCC^         G      -------        CU  GAUU    A  AU     A   AU      C        U     AG    AAA

(55) tsa-miR390a: AAGCUCAGGAGGGAUAGCGCC

AAA    --|     AU U  A          G           U  UU     AUU 
   GUAG  AGAAGA  C GU AAGCUCAGGA GGAUAGCGCCA GA  GUCAG   \
   CAUC  UCUUCU  G UA UUUGAGUCCU CCUGUCGCGGU UU  UAGUU   C
UAA    GU^     CG U  C          A           -  UU     ACA

(56) tsa-miR393a: UCCAAAGGGAUCGCAUUGAUC
      tsa-miR393b-3p:AUCAUGCGAUCUCUUUGGAUU


A      AAA                  U      .-UCAU|   C 
 CUAGAG   GGAUCCAAAGGGAUCGCA UGAUCC      UAAG U
 GGUUUC   CUUAGGUUUCUCUAGCGU ACUAGG      AUUC A
-      CUA                  -      \ ----^   A 


                                     UUUCCC     UGAU-      .-AAUAUUCUCA   G 
                                           AAUGA     AAUUUU            AUC A
                                           UUGCU     UUAAAA            UAG A
                                     CCUU--     UCGUU      \ ----------   A 


                                                                    
                                                             -   AA 
                                                              GGA  \
                                                              CCU  A
                                                             C   AA 
(57) tsa-miR395a: CUGAAGUGUUUGGGGGAACUC

G|  UC   UA         UG         U    AAU     UUU 
 AUG  UCC  GAGUUCCUC  AGCACUUCA UGGG   ACAUU   \
 UAC  AGG  CUCAAGGGG  UUGUGAAGU ACCC   UGUAG   C
-^  CC   CC         GU         C    AU-     UAU

(58) tsa-miR395b: CUGAAGUGUUUGGGGGGACUC

A   UCC   U         UU         U  -   A   ----|    AAAC 
 AUG   CCA GAGUUCCCU  AACGCUUCA UG UAA UAC    UCAGA    C
 UAC   GGU CUCAGGGGG  UUGUGAAGU AC AUU AUG    AGUUU    A
-   CGU   U         GU         C  A   A   UAUU^    AUUU

(59) tsa-miR396a: UUCCACAGCUUUCUUGAACUG

CUCUC     UC            C           CCUUUUUAAU       U   .-CUCAUAAAUC|    GC 
     UGUAU  UUCCACAGCUUU UUGAACUGCAA          AUUGGUU UAU            UAUUU  \
     ACAUA  AGGGUGUCGAAA AACUUGACGUU          UAGCUAG AUG            AUAAA  A
GACAU     GA            U           ----------       C   \ ----------^    AU 


                                                           ----       UA 
                                                               GUGAUUU  A
                                                               CGCUAGA  U
                                                           UCUA       UU 
            
(60) tsa-miR396b: UUCCACAGCUUUCUUGAACUU
tsa-miR396b-3p: GCUCAAGAAAGCUGUGGGAAA

CUG|    A                    A   U      CA      UUU     UUGAACCAAAACAAC 
   GUCAU UUUUUCCACAGCUUUCUUGA CUU CUUUUU  UUUCCA   UGUUA               U
   CAGUA AAAAGGGUGUCGAAAGAACU GAA GAAAAA  AAAGGU   ACAAU               G
AAA^    C                    C   -      AA      CUU     CUACAAUAUAUAAAA

           
(61) tsa-miR396a-3p: GUUCAAUAAAGCUGUGGGAAG


CUCUC     UC            C           CCUUUUUAAU       U   .-CUCAUAAAUC|    GC 
     UGUAU  UUCCACAGCUUU UUGAACUGCAA          AUUGGUU UAU            UAUUU  \
     ACAUA  AGGGUGUCGAAA AACUUGACGUU          UAGCUAG AUG            AUAAA  A
GACAU     GA            U           ----------       C   \ ----------^    AU 


                                                           ----       UA 
                                                               GUGAUUU  A
                                                               CGCUAGA  U
                                                           UCUA       UU 
          
 

(62) tsa-miR398a: UGUGUUCUCAGGUCACCCCUG

-| A    A     UA   A            CGCAAUCAAC GC      G   CC 
 GA CUCG CAGGG  GAC UGAGAACACAUG          G  UGUAAU AUG  A
 CU GAGU GUCCC  CUG ACUCUUGUGUAC          C  ACAUUA UAC  U
U^ C    C     CA   G            UUUUCUCU-- UA      G   UG

(63) tsa-miR399a: UGCCAAAGGAGAGUUGCCCUG

A       A        U      A        C  .-GACU|    UUU  U AU 
 GAGCAGU AUAGGGCA CUUUCU UUGGCAGG GA      GCAAU   GC C  \
 UUCGUCA UGUCCCGU GAGAGG AACCGUUC CU      UGUUG   UG G  U
C       C        U      A        A  \ ----^    UUU  U UU 


                                      GUUU   UA 
                                          GGC  \
                                          UCG  U
                                      GUC-   UU

(64) tsa-miR408: AUGCACUGCCUCUUCCCUGGC
tsa-miR408-5p: CAGGGAACAAGCAGAGCAUGG

UAAC|  AC  A       CAA    A      AUU   UUU  U      U  AA 
    GAG  AG CAGGGAA   GCAG GCAUGG   GAG   AC AAAACA CA  C
    CUC  UC GUCCCUU   CGUC CGUACC   CUC   UG UUUUGU GU  G
CUUU^  CC  G       CUC    A      CUU   ---  -      -  CA

(65) tsa-miR2111a: UAAUCUGCAUCCUGAGGUUUA

AA      ---       CC                    U      C   -|  U     AAAAAAAAAA    A 
  GUAUUG   GUGAGGA  GGGUAAUCUGCAUCCUGAGG UUAAGG UUA AUU ACGCA          AUGC U
  CAUAAC   CAUUCCU  UCCAUUAGGCGUAGGGCUCC AAUUUU AAU UGA UGUGU          UGCG A
G-      AUU       UC                    U      C   A^  U     GUAUUAAUA-    U 

(66) tsa-miR2111b-3p: AUCCUCGGGAUACAGAUUACC

---   CUUU     U   C          C         U        CUCUAA--|    U 
   GGU    GGUGA GAA GGGUAAUCUG AUCCUGAGG UUAAAACU        GCAUA A
   UCA    UCAUU CUU UCCAUUAGAC UAGGGCUCC AGUUUUGA        CGUAU C
ACG   UGC-     C   C          A         U        UCUGUAAG^    A

(67) tsa-miR400: UAUGAGAGUAUUAUAAGUCAC

-    A UG                          |     U  U 
 GAAG U  UUUAUGAGAGUAUUAUAAGUCACU--ACAUUU GG A
 UUUU G  AAGUACUCUCAUAAUAUUCAGUGA  UGUGAA CC G
G    A GU                        \ ^     C  A 


                                   U-    U 
                                     GUUU C
                                     CAAA C
                                   AG    C 
                                           

(68) tsa-miR403: UUAGAUUCACGCACAAACUCG

AGAA--|     U  U     AU    U                   AG  U-  UU   AU 
      GAAGAG CA AUUAC  GUUU GUGCGUGAAUCUAAUUCGA  GC  UA  AUC  A
      CUUUUC GU UAAUG  CAAA CACGCACUUAGAUUAGGUU  UG  GU  UAG  U
UUUUAA^     U  C     CU    -                   GU  UU  UU   CC


(69) tsa-miR827b: UUAGAUGACCAUCAACAAACG

UUU    --   C     U           U   -      U  A        -| CC 
   UGCA  ACC UUGAA GUGUUUGUUGA UGG CAUCUA GC AAUCGAUC AC  U
   ACGU  UGG AGCUU CGCAAACAACU ACC GUAGAU CG UUAGCUAG UG  C
UCU    UU   U     U           -   A      U  G        U^ UG

(70) tsa-miR845a: CGGCUCUGAUACCAAUUGAUG

G|     AUCA                    A   U  UUC 
 GUUUCC    CGUCGAUUGGUGUCAGAGCC CGC AA   U
 CAAAGG    GUAGUUAACCAUAGUCUCGG GCG UU   A
-^     CAAC                    C   U  UCG

The red means the mature miRNAs locating in the 5' of the pre-miRNA sequences, and the blue means the mature miRNAs locating in the 3' of the pre-miRNA sequences.
