# Supplementary material for: Genome-wide identification of Thellungiella salsuginea microRNAs with putative roles in the salt stress response
Source: BMC Plant Biol. 2013 Nov 15;13:180. doi: 10.1186/1471-2229-13-180 (PMC4225614; doi:10.1186/1471-2229-13-180)
Supplement: Additional file 8: Figure S2 — Hairpin structures of novel miRNAs of Thellungiella. [file 1471-2229-13-180-S8.rtf]

(1) tsa-miRn1: GGAGGAUGAUACAAGCUCUCAUA
   ¦¤G = -18.50 
    
  AUUA---------  AU  A      C    CG     CUUCG 
               GA  GC UGUAUC AUCU  UUCGU     \
               CU  CG ACAUAG UAGG  AGGUA     A
  AACCUUUCUAAUA  CU  A      -    --     CUAUC 
        

(2) tsa-miRn2: UAGGAAUCUGUGCUCAAACCAC
     ¦¤G = -57.10
   
G    UCC--                       -        UAC-|     C 
 AAAC     UGUGGUUUGAGCACAGAUUUCUA CAGGAGAU    CAUCUU U
 UUUG     ACACCAAACUCGUGUCUAAGGAU GUCCUCUA    GUAGAA A
-    UAUCU                       A        CUUU^     A 
       
(3) tsa-miRn3: UUGGGGGUAAGAGUAUUAUAC
tsa-miRn3-3P: UAUAAUACCCUUAAACCCAAU
     ¦¤G = -46.10
                   
GAG--|     U  CA   G          C    AA     UUAUUAGUUAUCG        CUU 
     AGGUUC CA  UCU UGUAUAAUAC CUUA  CCCAA             UAAUUGAG   U
     UCCAAG GU  AGA ACAUAUUAUG GAAU  GGGUU             AUUAACUC   G
UCUAA^     -  AG   G          A    GG     UUGUGCCUUA---        UUU 
        
                     
(4) tsa-miRn4: UGUUCGCCGGAAAAUAACCAG
      ÄG = -48.20
           
UG|      C                   G     -    UU 
  UUAAUCG CUGGUUAUUUUCCGGCGAA CAGAC GGUA  \
  AAUUAGC GACCAAUAAAAGGCCGCUU GUUUG CUAU  G
AG^      A                   -     G    UU 
         
(5) tsa-miRn5: UUUUAAAACUGAAAACGUAAU
     ¦¤G = -43.80
                
A      ---         A               .-AU|       U 
 UUGUAU   UAUUACGUU UCAGUUUUAAAACAU    UGUGUUGU U
 AACAUA   AUAAUGCAA AGUCAAAAUUUUGUA    ACACAACA U
-      UUA         A               \ --^       U 
           
 (6) tsa-miRn6: AUUGGAGUUGAUAUAAUGCAG
¦¤G = -70.70 
AUG                                   .-C    .-AAAACCGAUUU|  C   AAA  UUA 
   CUGCAACCUGCAUUAUAUCAACUCCAAUUCAAGUU   UUGA             UCU AGG   UG   C
   GACGUUGGACGUAAUAUAGUUGAGGUUAAGUUCAA   AACU             AGA UCC   AC   U
AUA                                   \ -    \ -----------^  C   CAA  UAC

 (7) tsa-miRn7: CAAGGCAGAAGAAGGCUGUUU
      ¦¤G = -63.10
                    
G|       U     C      A                      G 
 CAAAGUGA CAAGG AGAAGA GGCUGUUUUGGCAAUAGGAUUA C
 GUUUCAUU GUUCC UCUUCU CCGACAAAACCGUUAUCCUAGU G
A^       U     U      -                      C 
  
 
(8) tsa-miRn8: AAUGAAUGAUGCGGUAGACAAAU
tsa-miRn8-3p:UAGCUUCCGACUCAUUCAUCCA
      ¦¤G = -35.50
                   
A------|    UGAU           AG-  U   AC        C  AA  C      ACCAAGAA 
       GGUCG    AUGAUCCAAUU   CU CCG  UCAUUCAU CA  UA CGAGUC        \
       UUAGU    UACUGGGUUAA   GA GGC  AGUAAGUA GU  AU GUUCAG        U
GUCUCUC^    ----           ACA  U   GU        A  AA  U      AUCGAGCU 
    

£¨9£© tsa-miRn9: AGGAGAGUUUUGGUGUAGCAA
        ¦¤G = -29.80 
                 
CUACUUC     U      U        -   CUU---    AUAU    .-CC|     G 
       UUAUU CUAUGC CAGAACUC CUU      GUCA    AGUG    AUUUUG \
       AAUAA GAUGUG GUUUUGAG GGA      UAGU    UCAC    UAAAAC A
ACAAAAU     C      -        A   UACUUU    CUUU    \ --^     U 
     
 (10) tsa-miRn10: UGAAUUUGAUUUUAGACAGGA
      ¦¤G = -57.10
                  
-    UG  G                      U  U   .-GAU|        UCA 
 UGUU  UA CUGAAUUUGAUUUUAGACAGGA AG AUG     GAAAUCAAG   G
 ACAA  AU GACUUGGACUAAAGUCUGUCCU UC UAC     CUUUGGUUC   A
U    GU  G                      U  C   \ ---^        CCA 
  


(11) tsa-miRn11: UGAGUCGUCAAUCAGUAAGGU
     Tsa-miRn11-5p: AUUACUUGUUGACGAUUCCUU
      ¦¤G = -34.90
                       
UCA--|    U    A      A     U           C     UUA 
     AUUAA AUCA UUUGAC UUACU GUUGACGAUUC UUUGG   G
     UAAUU UAGU AAGCUG AAUGA UAACUGCUGAG AAAUU   A
GAGCA^    U    A      G     C           U     UAG 
      
 (12) tsa-miRn12: UGAUAGCAGUAGUUUCGUCUA
      ¦¤G = -23.00
         
C   UU   GUGA      U       -   .-AU|    U     -    CGCC 
 UUG  AUC    UAGCAG AGUUUCG UCU    GAGUG CAUAU AGAU    G
 AAC  UAG    GUUGUU UCGAGGC AGA    UUCGU GUGUA UCUA    G
C   UC   AG--      -       U   \ --^    -     C    AUAA 
     
(13) tsa-miRn13: UAGUGAAAUUGGAAAGUUGCC
      ¦¤G = -49.00
                    
AC|     A  A         U    C    U        G   CA       C   UA 
  UUGCAA CG CAGCUUUCC GAUU CACU CUCAAUUU ACC  AAAAUCU UGU  \
  AACGUU GC GUUGAAAGG UUAA GUGA GAGUUAAG UGG  UUUUAGA ACG  G
UU^     A  C         -    A    U        G   --       -   UA 
           

(14) tsa-miRn14: UCAUGAAGGAUCUGAGAUUGA
      ¦¤G = -47.00
                 
-|   A              AUC        A    A        CAUAG 
 GUUU CCAGUUCAUGAAGG   UGAGAUUG AGCU AAGAAGCC     \
 CAAG GGUCAAGUACUUCU   ACUCUGAC UCGA UUUUUUGG     A
C^   -              CAU        C    C        CCACA 
                 
£¨15£©tsa-miRn15£ºCUAGGGUUCCAGAAUUGAGGCUA
       ¦¤G = -25.90                  
U  A     U-|   GG    A           AAAAUGAC    GGC 
 CC UGGUC  CUAG  UUCC GAAUUGAGGCU        UUUG   \
 GG GCCGG  GGUU  GAGG CUUAAUUUCGG        AAAC   U
U  C     CU^   --    -           GUGU----    ACU 
    
£¨16£©tsa-miRn16£ºUGUGAUCCUCAGAUAGACGUACA 
       ¦¤G = -38.30                            
AUUCU    .-A|     C   A      C             GUUUA  U      AAUA 
     UCUC   UGUGAU CUC GAUAGA GUACAAAAAAUAU     UA GAAAGA    \
     AGAG   ACACUA GAG CUAUCU CGUGUUUUUUAUA     AU CUUUUU    U
UAUUU    \ -^     A   -      A             AUAC-  C      CACA 
          
£¨17£©tsa-miRn17£ºACCCCAAACCAGCUCAGACAA
       ÄG = -66.30
      A        U       C    UA      A   -|        U    GC   AUCCAA        UUAAGUUU     U  AG 
 UGAAGAAG AUUGUCU AGCU  UUUGGG UUG GAGGUCAAG CUAA  AAG      GAGGAAGA        GAACG GA  \
 ACUUCUUC UAACAGA UCGA  AAACCC AAC CUCCAGUUC GAUU  UUC      CUCCUUCU        CUUGU CU  G
-        U       C    CC      C   G^        C    --   ------        U-------     U  AA 
     
(18) tsa-miRn18: UGGAUUUAGAAUAAUGGUGGCUA 

ÄG = -47.30 
 
          A----|    AGAGAAAGUA      G  ACA        AAAA 
               AGCUA          UAGAUU AA   GAGAAGAA    \
               UCGGU          AUUUAG UU   CUCUUUUU    A
          AAACA^    GGUAAUAAG-      G  C--        GCAA 
  

(19) tsa-miRn19: AAGGCUGUGAAUUGUUUUGGC
¦¤G = -56.80                 
-                                G   UGGAA--|   AAA 
 UGUGUUUCUCAAGGCUGUGAAUUGUUUUGGCA CUA       CCUA   G
 ACACAAAGAGUUCUGACACUUAACAAAGCCGU GGU       GGGU   U
U                                -   UUAAAGC^   GGU 

 (20) tsa-miRn20: AAAGCAUGUGAGUACUUCGUA
tsa-miRn20-3p: TGAAAUAUCACAUGCUUUCG
¦¤G = -71.20 
                
UCA--     G   -                      C    U  CU--      CAA     -|     AACG 
     CUCCU GCU UGUUUGUGAAAGCAUGUGAGUA UUCG AG    ACACUC   GCCUG GAUGCA    G
     GAGGA CGA ACAAGCGCUUUCGUACACUUAU AAGU UC    UGUGAG   CGGAC UUACGU    A
UUACG     -   U                      A    C  UAUU      UCA     U^     ACUG 

     U        \ --------^    UG

£¨21£©tsa-miRn21£ºUGAAAGGAAACAUUGAUGUUU
¦¤G = -48.50 
AG--       C A      A-     A                 U--|     AUUC        CA 
    UCGUAU G AUAUUU  AAACA CAGUGUUUUCUUUCACA   AGUCUU    CAUGGAAG  G
    GGCGUG C UAUAAA  UUUGU GUUACAAAGGAAAGUGU   UCGGAG    GUACCUUU  C
UCAA       U -      AC     A                      UUU^     AUU-        UU

£¨22£©tsa-miRn22£ºUUGUGCAAGACUGAGAAGCAA
¦¤G = -57.50 
-|  U    U           CU      C          -       UAU 
 ACC CCGG GUUGUGCAAGA  GAGAAG AAAGCCUGUC UAUGUAU   G
 UGG GGUC CAACACGUUCU  UUCUUC UUUCGGACAG AUAUAUA   A
A^  -    -           CC      U          G       UAG


(1) PC001: UCGUAAUUACCUGAGACGAUAA
   ¦¤G = -172.40 
             
     
-             U                    U   U  CUC-----  A        .-C             CC----|                                 C        C  A-    AUUGA  CCA 
 UUCAUCAUCCUCG AAUUACCUGAGACGAUAAUG UGG GG        UC GUUCAUCU   CUGUGUAAGCCCA      GAGCACAGUUCAUGACGGUAAAAGAACACGUCGC GAUCUCAA AC  AGGG     AC   \
 AAGUAGUAGGAGU UUAAUGGACUCUGCUGUUAC ACC CC        AG CAAGUGGA   GACACAUUCGGGU      CUCGUGUCAAGUACUGCUAUUUUCUUGUGCAGCG CUAGAGUU UG  UCCC     UG   C
G             -                    U   C  CCCCCCAU  C        \ -             CAGACU^                                 A        -  GG    AUUUA  AAU 
      
 (1) PC002: AAGGAUUACAUAUUUGUUACACU
  ¦¤G = -56.50
     
UAAAUUCA     A     UU      GUC    .-AAACCGACGCAA   .-GUUUAACAAAAAGA|    AU 
        ACGGU GUGAU  GAUGUG   UCCU              GGU                UAAGA  \
        UGUCA CAUUG  UUAUAC   AGGA              CCA                AUUCU  C
CUUACUAA     -     U-      AUU    \ ------------   \ --------------^    AC 
                                                 

 (3) PC003: GCACCAGUGGUCUAGUGGUAG
¦¤G = -24.00 
          
GAGUAC      ----    G  -    AG    AUAACAA   -      .-CAAAAAAAAAAAAAAG| GAU 
      GUACUA    ACUA UA AUUA  GGUG       UGC GUUAGC                  GU   \
      CAUGAU    UGGU AU UGGU  CCAC       GCG CAAUUG                  CA   A
CC----      AAGA    G  C    GA    GAA----   A      \ ----------------^ AUG 
.          
(4) PC004: GUAGUAGAAAUGACCAGAUGAUA
      ¦¤G = -19.80 

           
UCCAGAAAAA    U     U  C--|      UAAAAAU    A   U   AAUAAA     U 
          GUAG AGAAA GA   CAGAUGA       AGAA UUA GAU      UUAUC A
          CGUC UCUUU CU   GUCUAUU       UCUU AGU CUA      AGUAG G
CUAUACUCGC    C     U  AUA^      UUGGGAU    -   -   ------     C 
.       

(5) PC005: CUGGCCCUGGAAGAGAAGAGAUA
     ¦¤G = -26.90
                   
       GAAAAA       -   AA  CUGA    U  GUU 
             UAUCUCU CUC  CC    CCAG AG   \
             AUAGAGA GAG  GG    GGUC UC   U
       UAUUAC       A   AA  UCCC    -  ACC 
       
 (6) PC006: GUGUAGAUAAUGCAAGACUCAUA
     ¦¤G = -85.80 
            
AAUGCAAAAA  G     A    AA     .-AUAAAAGAAAAGUGGUAAGAAAGGG  G      ---   -   AA------    AA-     AGUG   -   .-GUGUGAAA    -     -|  GG   AGAA 
          GU UAGAU AUGC  GACUC                           AU AAAUCC   UCC CCU        ACUU   AUCAC    UCC UCA          CAAG UCGAG AUU  AUG    \
          CA GUCUA UACG  CUGGG                           UG UUUGGG   AGG GGA        UGAA   UGGUG    AGG AGU          GUUU AGCUU UAG  UAC    U
CUUUUACUAG  A     C    GG     \ -------------------------  G      GUA   U   GUAAGUAG    GUG     GA--   U   \ --------    A     A^  A-   GUAA 
             

(7) PC007: GAAGACAAUGUAAAUAUUCGGUA
      ¦¤G = -42.10 
                  
A     GUAA      A    AAA      G   AA      .-AAAUA      .-A     -|       UUUU     UA 
 AAAGU    GAAGAC AUGU   UAUUCG UAA  UAAAUU       GUUUUU   UAAUC UUAUAAAA    AAGUU  \
 UUUCA    CUUCUG UACA   AUAAGC AUU  AUUUAA       UAAAAA   AUUAG AAUGUUUU    UUCAA  C
G     GA--      G    G--      G   AC      \ -----      \ -     U^       C---     UC 
    
 (8)PC008: AUCUGAUUUAUGACUAUGCGGUA
     ¦¤G = -29.90
             
U|   AA    G     C  U   A   A    C  UC   CC      AAA 
 UCAC  AAUU GCUGC UA UCA AAA UAGA UG  UAG  GCCUAA   \
 AGUG  UUAA UGGCG AU AGU UUU GUCU AC  AUC  CGGAUU   C
-^   GA    A     U  C   A   A    A  CA   A-      CGU 
        
 (9) PC009: GCUGACUGUGGGGAAUUACGGUA
      ¦¤G = -26.90 
                     
AUUU            UUUA    ----       --------|    U 
    GAAUAUUACUGU    CCCC    UUAGUGU        GAUAU \
    CUUAUAAUGGCA    GGGG    AGUCGCG        CUAUA A
AAAU            UUAA    UGUC       GUUAACAA^    U 
      
 (10) PC010: AGACUAGAACAAGCCGUUGAUA
     ¦¤G = -48.70
               
-    G    C   C     CA                C    -    -| UGU 
 AUAU UCAG AGA UAGAA  AGCCGUUGAUAUGCCU UUGA UAGC CA   \
 UAUA AGUC UCU AUCUU  UUGGCAACUAUACGGA AACU AUUG GU   U
C    G    C   A     CC                C    C    U^ CUU 
 
(11) PC011: AGAGAUCUCCAGUGGUUGGCUCA
     ¦¤G = -89.60 
         
UGUAGCAA        UGUC       -   AG  .-AUUCGUAC     CAUU--         .-GAUGUACGAUUUG    CU     C     .-AA|     AAU     AAUU 
        CUUGGGUC    CCGUUGG GAU  CU          UGGUG      ACAAGCUGG               GUUA  UUGGU GGGUC    CAAAGG   CUGGC    \
        GAACUCGG    GGUGACC CUA  GA          ACCAC      UGUUCGACC               UAAU  AACCG UCUAG    GUUUUC   GAUCG    G
AUUCUAUA        UU--       U   GA  \ --------     UCUGUU         \ -------------    UU     U     \ --^     ---     AUUA 
                              
 (12)PC012: AGAGUUGAGAGACAGGGACGACA 
     ¦¤G = -20.50
UA   A  -  .-AGA|  G 
  UGG GC GC     GUU A
  ACC CG CG     CAG G
UG   A  A  \ ---^  A 


                     
             .-A  -      ACUUU 
                GG GACGAC     \
                CC CUGUUG     G
             \ -  A      CGCGA 
                 
   
               U   U 
                UCC A
                AGG C
               -   A 
 
   
 (13) PC013: GAAGAUUGGCAUGAUUAAUGAUA
¦¤G = -21.40
                       
  AC----------   C       A       C      A CUU 
              UCA UAAUCAU CUAAUCU UCUUAA C   U
              AGU AUUAGUA GGUUAGA AGAGUU G   G
  GAGGUAAAGAAU   A       C       -      - AAC 
      
 (14)PC014: AUAUGUACGCAACUAUAUGGAUA
     ¦¤G = -57.60
                 
G      CAUAUA            G    C     AU                 .-CAU|            UAU 
 GACAUG      UAUCUAUAUAGU GUGU UAUAU  AUACAGCUAUAUGGAUA     AUAUAAUUAUAUA   G
 CUGUAU      AUAGGUAUAUCA CGCA GUAUA  UGUGUUGAUAUAUUUAU     UAUAUUAAUAUAU   U
-      ACAC--            A    U     GC                 \ ---^            CCA 
 (15) PC015: GAUGGUCUGCCUACGCUUCUAUA
      ¦¤G = -60.70 
                 
AAGCAUGA    U    UG    C     U   .-UAAAC      .-UAC   U  .-UUUU|     A   UC    CA 
        CCGA GGUC  CCUA GCUUC AUA       GGAUUG     UUG CA      GAGGAC UCA  UUUU  A
        GGCU UCAG  GGAU CGGGG UAU       CCUAAC     AGC GU      CUUUUG AGU  AAAA  U
UCACAAAG    U    --    U     C   \ -----      \ ---   U  \ ----^     -   UA    AA 
     

(16) PC016: UGUGAUCCUCAGACAGACGUACA
     ¦¤G = -41.00
                
AUUC|           C     A    C      A      GUUUACAU      AAAA 
    UUCUGAUGUGAU CUCAG CAGA GUACAA AAAUAU        GAAAGA    \
    AAGACUACACUA GAGUU GUCU CGUGUU UUUAUA        CUUUUU    U
GAAA^           A     -    A      C      AUACAUC-      CACA 
              
(17) PC017: AGAAGAUAUGUGGUGUUUUGAUA
   ¦¤G = -18.30 
                   
AAAAAAAAAA    GA-| U       U     A    UA 
          AGAA   UA GUGGUGU UUGAU AAAG  \
          UCUU   GU UACCAUA AACUG UUUC  U
GAUAGAUAUA    AUG^ U       C     A    UC 
.              
(18) PC018: AAUUGACAAAGUUAUGCCAGA
      ¦¤G = -56.00 
               
U    C    GAU             C          C   UAG       .-GUAAA|      C 
 CGAA UGCC   UUUUGGCAUAAUU UGUCAAUUCC UCA   AUGUAAU       GUUGGUU G
 GCUU ACGG   AAGACCGUAUUGA ACAGUUAAGG AGU   UACAUUG       UAACUAG G
-    U    ---             A          A   UUG       \ -----^      U 
               
(19) PC019: UUUGAAUCUGAGAAGCAAGUUUA
     ¦¤G = -61.20
             
UC    UUUC   U    U      U-    UUA----  UG -    U---    UCU-     C    UU   .-UUUGUACAU|    AAA 
  GUGU    UGA CUUG UUUCUU  GUUC       UU  A CUUU    GCAU    UGAGU UCAU  AGC           AUGAU   A
  CACA    AUU GAAC GAAGAG  UAAG       AA  U GAAA    CGUA    ACUCA AGUG  UCG           UACUA   U
CU    UUAA   U    -      UC    UUUAGCA  GU C    UGUU    UCUU     A    U-   \ ---------^    CGU 
                          
 (20) PC020: AUACUAAUAGGGAGUUGGACA
     ¦¤G = -30.20
              
AUAUAAGUUAAA         A-        .-GAGAUAU|   G 
            UCCAACUCC  AUUAGUAU         UUUG A
            AGGUUGAGG  UAAUCAUA         GAAC A
UAGUUUUUGUAC         GA        \ -------^   A 
                     
 (21) PC021: CAGGAGAAGUCUACUGAAGUCUA
     ¦¤G = -35.50
                    
-               --          .-GAAGUCUA|  G 
 GAGUAGACUUCAGGA  GAAGUCUACU          UUG U
 UUCAUUUGAAGUUUU  CUUCAGAUGA          AAC U
U               UA          \ --------^  A 

 (22) PC022: ACAACAGUGUUUUCUUUCACA
     ¦¤G = -41.60
             
A      A-     A                 U--|     AUUC        CA 
 AUAUUU  AAACA CAGUGUUUUCUUUCACA   AGUCUU    CAUGGAAG  G
 UAUAAA  UUUGU GUUACAAAGGAAAGUGU   UCGGAG    GUACCUUU  C
C      AC     A                 UUU^     AUU-        UU 
     
(23) PC023: AACUAAAGACAAAAACAAAGACA
      ÄG = -33.20
                
U   AUUCCU     AA        CA-     .-AAAUAUAUGUAAA    CCUAA      G    AGA-|   A       ACAAAA 
 GCA      AACUA  GACAAAAA   AAGAC               ACUA     AUGUAU CAUG    CUCA UAUUUGG      A
 UGU      UUGAU  CUGUUUUU   UUCUG               UGAU     UAUAUG GUAU    GAGU AUAAAUC      U
G   GACU--     CA        UUG     \ -------------    UUGAC      A    AAAA^   -       CUAACG 
    
       
(24) PC024: AGAUUAGGGUGCUUAGAUGUUUA
      ÄG = -50.60
     
                          
  U-------------     ---       .-UUGUAU      -           U    U    ---    G 
                CAUCU   UACCUUA        UUACUU CUAAUCAUGAU AACA CACC   UCUU C
                GUAGA   GUGGGAU        GAUGAG GAUUAGUACUA UUGU GUGG   AGAA A
  GUAGUAUUUAAUUU     UUC       \ ------      U           C    -    UAA    A 
                                UA
(25) PC025: UACCGACUGCCUACUUAAAUAUA
      ÄG = -23.40
              
A  GAAAA          U-   --  U    .-CU|     U    UAGAUCAAU  A          G 
 UC     AAUUAUAUUU  GUA  UA UCGG    UGGAAU GCAA         GU GAAGUUUUUA U
 AG     UUAAUAUAAA  CAU  GU AGCC    ACUUUA UGUU         UA CUUUAAGAGU U
A  AAUG-          UU   CC  C    \ --^     U    ---------  A          U 
  
 (26) PC026: AGAAGGGACGCCUCGUUGUUUUA
      ÄG = -52.30
             
A     UUGG--       G     -   UC  .-CA    U-| UA   A     AA 
 GGAUU      AAGGCAA GAGGU UCU  UC    AGGU  UU  ACG GGCAG  A
 CUUAA      UUUUGUU CUCCG AGG  AG    UUCA  GA  UGC UCGUU  U
-     UUUUAA       G     C   GA  \ --    UU^ GC   A     AC 
                                  A
 (27) PC027: GAUACCGCGUUGGAGAUGGCCUA
     ¦¤G = -24.10
                    
U       AU     C-     G  GAU   C--      .-G|    UGA 
 AUCGCGG  GAUAC  GCGUU GA   GGC   UAAUAA   GUAAA   U
 UAGUGUU  UUGUG  CGUAA UU   UCG   AUUGUU   CAUUU   U
C       --     UU     G  ACU   UAC      \ -^    UGU 
    
(28) PC028: UAUGUUGCAUGGGAUUAUCAAA
      ÄG = -33.70
              
-| CA    C        CA       A       A     U 
 GU  UAAA AUAUGUUG  UGGGAUU UCAAAAC GUUCA A
 CA  AUUU UAUACAAC  ACCCUAA AGUUUUG CAAGU U
A^ AC    A        AA       -       A     U 
          
 (29) PC029: UCAGAUCAUGUGGUAGUUUCA
      ¦¤G = -43.50
                     
CAG      A           G        AAA       C-  ---| GC 
   CAUCUG UGAAGCUGCCA CAUGAUCU   CUUCUCU  UC   UC  U
   GUGGAC ACUUUGAUGGU GUACUAGA   GAAGGGA  AG   AG  C
UUA      C           -        CUA       CU  UUU^ AU 
       
 (30) PC030: AGAGGAGAGUUUUGGUGUAGCUA
      
     ¦¤G = -28.60
          
CUAC-   GUAUGU  A   -         -    .-UU   UU   AG  --| AU 
     UUC      GC UGC UCAGAAUUC CCUU    GUC  UAU  UG  CC  \
     AGG      CG AUG GGUUUUGAG GGAG    CAG  AUA  AC  GG  U
UAAUA   AAAU--  -   U         A    \ --   U-   AA  UA^ UU 
                                    A
 (31) PC031: AAUUGAUGUGAGAUAACCGGA
      ¦¤G = -47.40
          
G        AGA          A           A   C    .-UUAA  -|   C    A 
 GCUAAACC   CCGGUUAUCU AUAUCAGUUUA UGU AUGU      GU GUAA ACCA A
 UGAUUUGG   GGCCAAUAGA UGUAGUUAAAU ACA UACA      CA CAUU UGGU A
-        CCA          G           C   A    \ ----  U^   -    G 
            

(32) PC032: GUGGAAACUGGCAUACUCCAA
      ¦¤G = -53.60
                   
GA          C  U   CACC    A          .-CUCACCAUCCGUACC    A    AAC--    GU-|  G 
  CAUGUUGGUU GA UGU    GUCA UUUUUACUAC                 GGAG AACA     GGUU   UUC A
  GUAUAAUCAA CU AUA    CGGU AAAGGUGGUG                 CCUC UUGU     UCGA   AAG A
CC          C  C   ----    C          \ ---------------    C    GCUCU    AAU^  A 
       

 (33) PC033: CAGAGGAAGAAGAAUGUUUGCUA
¦¤G = -61.40 
GUCAC    A       .-A  AG     U-  .-CUA|     AUCCA 
     CAAU CAGAGGA   GA  AAUGU  UG     GAAAAU     G
     GUUA GUUUCCU   CU  UUACG  AC     CUUUUA     G
UCUUC    -       \ -  CU     UU  \ ---^     AUGCU

 (34) PC034: AGGAGAGUUUUGGUGUAGCUU
      ÄG = -34.50 
         
             UACUUUUUAUUAG     U        -   .-CU  U  AU 
                          UAUGC CAGAACUC CCU    UG CA  A
                          AUGUG GUUUUGAG GGA    AC GU  U
             UUCG---------     -        A   \ --  C  GU 
   


 (35) PC035: AUGAGUUGACGUGUGCGUGGAUA
        ¦¤G = -27.90 
                
U    AAA-|      UU   -   AAG         CG-   UU 
 CACG    CUUAUCC  UGC CAU   UAAUUCGUG   GAC  A
 GUGU    GAAUAGG  GCG GUG   GUUGAGUAC   CUG  A
-    AAAA^      U-   U   CA-         AAA   UC 
           

(36) PC036: GGUGAACUUCUGACGGUUUGACA              
      
 ÄG = -86.40
  
 
    AAAU  G    -  A-      G  G     ---     CA 
        UU AGCU GG  UCAGAC GU AGAAG   GCCUU  A
        AA UCGA CC  AGUUUG CA UCUUC   UGGGG  U
    ----  G    G  AC      G  G     AAG     AC 
 
 (37) PC037: UAACGAGACAGAAAUCAUUGCUA
       ¦¤G = -34.60
                            
G       ----  UG    -      A   UUUU----      U     ----|    GA 
 UAACUUA    GG  AUGA UUCUGU CUU        CCUUGG GAGGA    UUUUG  \
 AUUGAGU    UC  UACU AAGACA GAG        GGAACC CUUCU    GGAAC  A
-       UGCA  GU    A      -   CAAUUUUU      U     UGGA^    GG 
 .               
 (38) PC038: GUCUGGGUGGUGUAGUCGGUU
 
      ¦¤G = -53.00
              
-   CA     G   G    G         .-UUAUCACG|    CUC 
 GAG  UCAAC UCU GGUG UGUAGUCGG          CUAGU   \
 UUC  AGUUG AGA UCAC AUAUUAGCC          GAUCA   A
A   AG     -   A    A         \ --------^    CAC 
 .         
 (39) PC039: AUUCGACGUGGAUAUACAUUUUA 
      ¦¤G = -82.60
 
-        A       C         C                   A       A  UG        U   .-UUC|    UA 
 UCAUUCAU UAUUCGA GUGGAUAUA AUUUUAUAUUGUCUAUAAU UGGACAU AU  UGUGGAUA GUU     AUUGA  \
 AGUAAGUA AUAAGCU CACCUAUAU UAAAAUAUAACAGGUAUUA ACCUGUA UA  ACACCUAU CAA     UAACU  G
A        A       A         A                   A       C  GU        C   \ ---^    UA

 (40) PC040: GAGCUGUGAAGAAUUUGGCU
ÄG = -24.30 
G|    GUAC  AC        GA   C     G  GC 
 CCACG    AG  CCGGGUUC  UUC CGGCU GU  \
 GGUGC    UC  GGUUUAAG  AAG GUCGA UA  A
G^    GACU  --        --      U     G  AG


 (41) PC041: UAAACUUAUAGAGGUCUAAGACA
      ¦¤G = -76.20
                
CCA      GU-   AUUC     GAUU        --   G    UU       U           -        .-UG         ---|    G 
   UAAAGU   UCU    UCUCU    AUGAGUUU  GCA UGGU  CUGUAUA UGUU--CUUUU AUUCAUAU    UUAAUUUUU   UUGGU G
   AUUUUA   AGA    GGAGA    UAUUCAAA  CGU GCCG  GACGUAU ACAA  GAGAA UAAGUGUA    AAUUGAAAA   AACCA U
UAA      AAC   AUCU     ----        UU   -    U-       C    \      C        \ --         ACU^    C 
.       
 (42) PC042: AAGAGACCAAUUCGGUAAGAA
      ¦¤G = -53.70
                        
UGA       CUC     -  U-   C           CUA    .-AGA   U       .-AA        UAAU--       --|      AUUC 
   AUGGAGG   UUCUU CC  GUU GUCUUUUGCUU   AAAC     UAA UUAUAUU    AAAAUAAA      UUAUAUA  AAAUUUG    \
   UACUUUC   AAGAA GG  UAA CAGAGAAUGAA   UUUG     AUU AAUAUAA    UUUUAUUU      AAUAUAU  UUUAGAC    U
GAA       ---     U  CU   C           ---    \ ---   C       \ --        UUUUUU       UU^      AAAA 
 .                 
 (43) PC043: AUCGGAGAUUUGAGGUGUAUCUA
       ÄG = -18.60
               
CUGAUUUUUCA|     AUUU          UAA---   GAAUU 
           UCGGAG    GAGGUGUAUC      AGG     \
           AGCUUC    CUCUACGUAG      UUC     A
UGAUGAUUUAC^     ----          UAAAGG   AAAAG 
          
(44) PC044: AAUGUGGAAGGAGAUGCAUCAUA
      ¦¤G = -56.10
          
U         CA  A        A-                .-AU| AU 
 AAAUGUGGA  GU GAUGCAUC  UUUCCAU--GUUUCUA    UC  \
 UUUGUACCU  UA CUACGUAG  GAAGGUG  UAAAGAU    AG  U
U         A-  -        AG       \        \ --^ AC 
           
£¨45£©PC045£ºAAUGAUAUUCGUGUCCGUUGA
       ¦¤G = -22.50
              
UA    GCC          C    CCG    C  .-AGUAAUUUACUUU|    CU 
  GUAC   AAAUGAUAUU GUGU   UUGA CA               UAGAG  \
  CAUG   UUUGCUAUAA UACA   AACU GU               AUUUC  C
UC    GAA          U    AG-    A  \ -------------^    AG 
     
£¨46£©PC046£ºCAUACUGAUGAGCAUUGGCCCUA 
   ¦¤G = -49.00
          
AUUCCU      UA     G    UU  CC   .-UCAUUCUCU|  A  AA 
      GCUCCA  CUGAU AGCA  GG  CUA           CCA CA  G
      CGAGGU  GAUUA UCGU  CC  GGU           GGU GU  A
CCCGUU      C-     G    UU  UU   \ ---------^  -  CC 
                          

£¨47£©PC047£ºAGAUGAUAUCAUGUAGUAUAGUU      
ÄG = -38.30    
AA     UUUUG   AU-          AA       AAAAUU           AU       .-AA|    UA 
  UUUUA     UAA   UAUUACAUGA  UGUCAUU      AACAAAAAAUA  UAAAAUA    AAAGA  U
  AAAAU     AUU   AUGAUGUACU  AUAGUAG      UUGUUUUUUAU  AUUUUAU    UUUCU  A
AA     UA---   GAU          --       ACU---           AU       \ --^    UA 
    
     
£¨48£©PC048£ºAUGACUUAAAACAGAAGCGCUUA
       ÄG = -69.20            
C U        CAC      ---    GAA  C      |    AAAGGAG     A  C   UU 
 G UCUCCUAU   AAGUGU   UGUU   GA UCAU--GCCUC       CUCCA GA UGA  \
 C GGAGGGUA   UUCGCG   ACAA   UU AGUA  CGGAG       GAGGU CU AUU  U
A -        A--      AAG    AA-  C    \ ^    -------     C  -   UU 
            
(49) PC049: UUAGAUUGUUUAAGUUUUGCAUA
¦¤G = -45.80
               
-   U     -         U      UUU-   UAAC--     .-AAGUGG|       CUU 
 GAA CAAGA UUUAGAUUG UUAAGU    GCA      GGAAA        ACUAGUAU   \
 UUU GUUUU AGAUUUGAC AAUUCA    UGU      CCUUU        UGGUUAUG   U
G   C     C         C      CUUU   UUUUAU     \ ------^       UGU 
  
(50) PC050: CAUGUAGAAAAGAGUGGUAGACA
¦¤G = -19.30 
              
CUGU---         GUA  A     U     --|   GACC   GAU 
       UAGAUCCAU   GA AAGAG GGUAG  ACAA    GGG   \
       GUUUAGGUG   UU UUCUU UCGUU  UGUU    UCU   A
AGAUCUU         GUG  G     -     AA^   AACA   ACC 

(51)PC051: CAGCUGGUUUACGGGACAUCUUA
¦¤G = -30.90           
-|   GCG        GG   A   G   UC    UC    C    CU  UG  UG 
 GAAU   UGCCAGCU  UUU CGG ACA  UUAC  UUUG GUAA  UU  AU  A
 CUUG   AUGGUCGA  AAA GUC UGU  AAUG  GAAC CGUU  AG  UA  A
C^   ---        --   -   G   GA    UC    -    --  GU  UC 
(52)PC052: CCGAAAUGUGGAAGGAGAUGCAU
ÄG = -60.00 
           
-  U     .-AUCC        G   GA        A-   A       UCUU    -    |   AU 
 AG AGAGA      GAAAUGUG AAG  GAUGCAUC  UUU CAUGUUU    AUUC CU--UUGG  A
 UC UCUCU      UUUUGUAC UUC  CUACGUAG  GAA GUGCAAA    UAAG GA  AACC  C
A  C     \ ----        A   A-        AG   G       UCC-    A  \ ^   CA 

 (53)PC053: UGUGGAAGGAGAUGCAUCAUUUG
ÄG = -57.90 
           
ACU       G   GA        A-           -------    AUU     AG-   CAA-      U       .-GAAA|      AAC  A    C   C 
   AAAUGUG AAG  GAUGCAUC  UUUCCAUGUUU       UCUA   CCUUU   AUC    CUCAAA CUCCUAU      AUCAGAA   UG GAUU UUA U
   UUUGUAC UUU  CUACGUAG  GAAGGUGUAAA       AGAU   GGAGA   UAG    GAGUUU GAGGGUA      UAGUCUU   AU CUGA AAU A
AUU       G   A-        AG           UCAUCAC    ---     AUA   AUCA      U       \ ----^      ---  A    A   G 

  
(54) PC054: UGGCGUCAGAAGAAGAGAGGAUA 
ÄG = -81.90
 
    ------------     -   G   .-AG             U-      GUUU        C 
                UCCUC CUU UUC    CGAGU--GCUUGU  UGGCUU    UUCCU--G U
                AGGAG GAA AAG    GCUUA  UGAACA  GUUGAA    GAGGA  C G
    CUAUAAAAAAAU     A   G   \ --     \       UU      AAAU     \  G 
                              ACUGCGGU

(55)PC055: UAAUUGGAAGAAUAUAUGUCUCA
ÄG = -30.90         
UAAAAAAUAA      GA         ---    .-CAAAUAAUAAAUAAUUC   A   --|    U 
          UAAUUG  AGAAUAUAU   GUCU                   AUU UGU  GAAGA U
          AUUAAC  UCUUGUAUG   CAGA                   UAA ACA  UUUCU A
GUAAUAAUUC      G-         UAU    \ -----------------   A   UC^    U 

 (56) PC056: AGAUUGAAAUCGAUGGAUGCAUA
ÄG = -33.30 
              
-        -      U     CGA GG U      .-AUUAUGGGGGAG|     GGGUG  UA    UUUU 
 CAGGGAGA GAAGAU GAAAU   U  A GCAUAA              UUUCUU     UU  CGGG    \
 GUUCCUCU UUUUUG UUUUG   G  U CGUGUU              AGAGGA     AG  GUCU    U
A        A      U     UAG UU -      \ ------------^     AUG--  GG    UAAA 
 (57) PC057: UGAACGGCCAUGUAGUGACAU
¦¤G = -29.70 
      
A|    GU    GC        A    C      G  GCUU  UUU  UC 
 AUUUA  UGAA  UCACUACA GGUC UUCACU CU    CA   GA  U
 UAAAU  ACUU  AGUGAUGU CCGG AAGUGG GG    GU   CU  C
-^    GC    AC        A    C      -  UU--  UCU  UU 

 (58) PC058: AGCGGAUCAAAGCAGCUACAC
¦¤G = -93.20            

U                                                 .-GAUAAAU| UC  A 
 CAUGUCUGUAGUGUAGCUGCUUUGAUCCGCUUAUGAGGUUCUGCUAUAA         GC  AG U
 GUACAGACAUCACAUCGACGAAACUAGGCGAAUGCUCCAAGACGAUAUU         CG  UC U
-                                                 \ -------^ UA  G 
  (59) PC059: GAACAUGAUUUAUGAAUUCGGCA
¦¤G = -85.90 
        
UUUAUAU     G    AAA     AU            U        --       ACGC     G     AAA           .-AUUA|  UA      AAA         AG     GA      AUCAA 
       UUUUG UGGA   AUAAA  UUAUGUUCUUGC UAAAAACG  AGAUUUU    UUUUG AAGAA   UGAGAUUUUAU      UUU  UGGGAA   GAGAUUUUA  UUUUU  CAGAAA     G
       AAAAC GCUU   UAUUU  AGUACAAGAGCG GUUUUUGC  UUUAAAA    AAGAC UUCUU   AUUUUAGAGUA      AAG  AUUCUU   UUUUAGAGU  AAAGG  GUUUUU     A
UUAUUUU     G    AAG     --            -        AU       CUA-     A     GC-           \ ----^  GC      GCA         AA     AG      GCAUU 

 (60) PC060: AGAGAUAGUUGGUUAUGAUCACA
¦¤G = -38.20
       GAAUA   A   G  UUA    U    CA     UCUUU   A       A   G 
            UUG UGA UC   AGCU ACUG  UCUUU     UCC GGGCCAG GGG A
            AAC ACU AG   UUGG UGAU  AGAGA     GGG CCUGGUC UCU A
       UGAC-   -   -  UA-    U    AG     UUU--   -       A   A 
  (61) PC061: UUCUAGGUGUGUAGAUUAGGGUA
¦¤G = -25.00 
          
.-UCUUUUA    U-  -|  UGUGU   UU     A  U  AUG     UAA 
         AGCU  CU AGG     AGA  AGGGU CU AG   UAGAU   U
         UCGA  GA UCC     UCU  UCUUA GA UC   AUUUG   G
\ -------    UU  U^  UUUCC   UC     -  U  GA-     UUA 
                   
(62) PC062: AGAUGCAUACUAAGGCCUGUCUA
ÄG = -28.00 
               
-         -    UGCA| -      C    .-CUAAAAUAAGAAAAACAA      AAUAG 
 CUUUUUUUU UAGA    UA CUAAGG CUGU                    AACUUU     \
 GAAAAGAAA GUUU    AU GAUUCC GAUA                    UUGAAA     A
G         C    CAG-^ C      U    \ ------------------      GAAAA


 (63) PC063: UGAGGUAGUUUGGCGUGUUGCAA
¦¤G = -46.20 
            
A|     UAGCUAC   AA     -           A   UUUUUC--           UUUUAU  CA 
 AGGCCA       AUC  GCAAC CGUUAAACUAU CUC        CCUUCAUAGAG      CC  \
 UCCGGU       UGG  CGUUG GCGGUUUGAUG GAG        GGAAGUAUUUC      GG  A
C^     -------   AA     U           -   UAAUUUUU           UUUU--  AG 

(64) PC064: UCGUUUAGCUCGACGUAGGCAUA
ÄG = -45.60 
            
UUGUU       GUUU    C  -  U           .-AUC   -       |    U      CCA 
     UUAAGUC    AGCU GA CG AGGCAUAAAAA     AGG CUGAA--UUAAA GGCACU   C
     GGUUUAG    UCGA CU GC UUUGUAUUUUU     UUC GGCUU  AAUUU CCGUGA   U
CUCCU       GUCC    -  A  U           \ ---   A     \ ^    -      AGU 
           
(65)PC065: AGUGACUUAUAAUACUCUUAU  
ÄG = -66.10 

UA       U                     .-AAAC|      CC 
  CCAAAUG AGUGACUUAUAAUACUCUUAU      AAUCUUU  A
  GGUUUGC UCACUGAAUAUUAUGAGAGUA      UUAGAAA  A
AG       U                     \ ----^      AG

 (66)PC066: GUGAGUGAUGUGCCAUAUUCUCU
ÄG = -29.90 
        10        20           30          40        50          60       
GUCUGAUCUGCUUC      ---   A  G     --  A      CUGA     A     .-UGAU|   UU 
              UGCAGA   GUG GU AUGUG  CC UAUUCU    UCCAA UUCAA      UGGU  A
              ACGUCU   UAC CA UAUAU  GG GUAAGG    AGGUU AAGUU      AUUA  U
AAAGUCUAA-----      GCA   -  A     UA  A      ----     A     \ ----^   UG 
   170            160        150       140           130              70  

 (67) PC067: UCCAGGUACCUUAAACGAUAU
ÄG = -58.73         
-   U    C    -      C  AAA         |     C UG UC  UC 
 CUU UGGA CUCC AGGUAC UU   CGAUAUU--AAUGUU U  A  GC  A
 GAA AUUU GAGG UUCGUG AA   GCUGUAG  UUACAG A  U  UG  C
C   C    U    U      C  ---       \ ^     U GU UU  UA 
     
(68)PC068: AGAGGUUUAGCAUUGGAAGAACA
¦¤G = -24.40 
        10        20        30          40         50         
UUCUGC     GUU    G   U   CACU      .-CAA    U-| C     UUCUUU 
      UUAUU   UUCU CCA UGC    GACCUC     UGUG  AG UGAAU      \
      AAUAA   AAGA GGU ACG    UUGGAG     AUAC  UC ACUUG      G
GUUCA-     AC-    A   U   AU--      \ ---    CC^ U     UUCUUC

(69) PC069: GUCAAUUGGUGGGAGAUAGG
ÄG = -50.40   
.-UAGUU   U                A    A--       .-AUUUAUAUUC UG AG   -|   CA 
       AGA UGUCAAUUGGUGGGAG UAGG   GCUUAUG            G  A  UUG GGUC  A
       UUU ACAGUUAACCAUUCUC AUCC   UGAGUAC            C  U  AAU CUAG  A
\ -----   U                -    AAC       \ ---------- GU AG   A^   AG

 (70) PC070: UGAAGGAUCGAGGUCGAGGCA
 ÄG = -22.20 
AAUAACAA         U    ----------|    GGC 
        GGUGAAGGA CGAG          GUCGA   \
        CCACUUCCU GCUC          UAGUU   A
CAACGUGG         -    GACUUAGAUA^    AUG 
.                
(71) PC071: AAUGAGAUUUUACGUUUUUGGCA
¦¤G = -59.90 
GAGCAA        -   U   A  -       C      -          AC     UA  .-G      .-AAAAUUUU   CU     ACAGAGAA--|     A  UU   CG     G 
      UGAAAAUG AGA UUU CG UUUUUGG AAGAAA AUGAGAUUUU  GUUUU  CG   AAAAAC          ACG  UUUUG          AAAACG AA  UUA  UUUUU A
      AUUUUUGC UUU AGA GC AAAGACC UUUUUU UAUUUUAGAA  CAAAA  GC   UUUUUG          UGC  AAAAC          UUUUGC UU  AGU  AAAAA C
AGAGCA        A   U   -  A       A      A          --     GG  \ -      \ --------   UU     AAAAGGGAAG^     A  UU   AU     A

(72) PC072: GGGAUUGUUUCUAGAGAAUGGUA
¦¤G = -38.20            
-      --       -       C        G   ------|   UAU        .-ACUUUUGAAACAUUUUUUUAACCAAUA          GAUUAU 
 UCCUAU  UUUUGGG AUUGUUU UAGAGAAU GUA      AAUA   UUUUGGAU                             AAUGAUAUUU      \
 GGGAUA  AAAAUCU UAAUAAA AUUUCUUG UAU      UUAU   AAAACCUA                             UUACUAUAAA      U
U      AU       G       -        G   AAUAUU^   CUU        \ ---------------------------          AAUUUU 
  
 (73) PC073: UGGAAAGUGAACAAAAGAUGA
¦¤G = -30.80            
AAUAUG     G        A  U      .-UUUUAUGUUUUA|  UG  UU   GG 
      CUUUU AUCUUUUG UU CUUUCU              GGA  UU  GAU  G
      GAAAA UAGAAAAC AG GAAAGG              UCU  GA  UUG  U
AGUAAA     G        A  U      \ ------------^  GU  U-   UU 

(74) PC074: GUGGAUCUGUUGUUCAAGAGGUU
¦¤G = -74.50           
CUAA    AAGA            U      GA-  UA---   A       UU  .-UAAGUGCAU      .-CA| GAU      GUC 
    CUCA    AACCUCUUGGGC AAUAGA   AU     UCC UUGAAGG  GG           AGUCCC    CU   UUCAUU   A
    GAGU    UUGGAGAACUUG UUGUCU   UG     AGG GAUUUCC  CC           UCAGGG    GA   AAGUAG   U
ACCC    AG--            -      AGG  UUUUA   C       U-  \ ---------      \ --^ AC-      AAG 
 
 (75) PC075: AAUCUGUGUGCAUAAUCUUAA
 ÄG = -54.40 
              
.-AUAUUUUA    U     UG AU-        --    A      .-AAUAA|   GG 
          UUAA CUGUG  C   AAUCUUAA  AACG CAAUUA       AUUC  \
          AAUU GGCAC  G   UUGGAAUU  UUGC GUUGAU       UAAG  A
\ --------    -     CU GUU        GA    A      \ -----^   GG 
              
 (76) PC076: AUAAGGAUGUGUCAUACUUUUUA
 ¦¤G = -26.50               
-    A  A         U     .-AUACUUUUUAAU|      A     UU    U 
 GAGA UU AUAUAAGGA GUGUC              UUAAGGU UUAUU  UUUU U
 UUCU AA UAUGUUUUU UAUAG              AAUUUUA AAUAA  AAAA U
G    C  A         -     \ ------------^      -     UU    U 
 
(77) PC077: AGAAGAAAUGGAAUGAUGUAUUU
 ÄG = -32.30 
-    U--        .-AGA          .-UGUAUUUC       U   UAUU   -|     U      UUU  AUA        A     UUG 
 UCAU   UAGAAAGA     AAUGGAAUGA          AAAAAAU UUA    AUA UAAUAU UAUAAC   UG   UAUUUAAA UAAAU   A
 AGUG   GUCUUUUU     UUACUUUAUU          UUUUUUG AAU    UAU AUUAUA AUAUUG   AC   AUAAAUUU AUUUA   A
G    CCU        \ ---          \ --------       U   UAUU   A^     C      UUU  ---        -     UUU 
          
(78) PC078: AUAAACCUGAAGCACAAAAGACA 

ÄG = -26.80 
             
            GA    C-    U     U    CC   AU 
              UGUC  UUGU GCUUC GGUU  AUG  U
              ACAG  AACA CGAAG CCAA  UAC  A
            AA    AA    -     U    A-   GU 
                 
 
(79) PC079: ACUUGAGGAGAUUAGGCUUGGG

ÄG = -100.80         
AUAAGGU      U--       A       AC     .-GC          .-UAUC|     A  GA      C 
       AGCUUC   CCUGAUC UCCUCAA  UAGUG    CAGGUUGAAG      UAAUAG CC  UUAAGA C
       UCGGGG   GGAUUAG AGGAGUU  AUUAC    GUCUAACUUU      AUUGUC GG  AGUUCU A
AGAGUUU      UUC       -       CA     \ --          \ ----^     A  AG      U 
             
(80) PC080: UAAUGGAAUUGGGGAGUCAAG
ÄG = -44.50 
        
U                 UU   UU           .-A    -| UUGUCUA 
 AGCUUUAUUACUUGAUU  CUA  UUUCAUUAUUU   UUGG UC       \
 UCGGGAUAAUGAACUGA  GGU  AAGGUAAUAAA   AACC AG       C
-                 GG   U-           \ -    U^ UUAAUAA 
             
(81) PC081: AUUCCGGAACCCUAGAGACCAUA
¦¤G = -62.80 
         
-  C    U      C   ACC-  A    C     .-AG        -   AUAAAU     UCAUU      .-GUUUUU|   A 
 CU AGCC CAAUUC GGA    CU GAGA CAUAG    UAUUGAAG ACU      ACUUG     UAGGUC        AGGG C
 GA UCGG GUUGAG CCU    GA CUCU GUAUC    AUAACUUC UGA      UGAAC     GUUUAG        UCCC A
A  U    -      C   AGAU  -    -     \ --        U   GCC---     UU---      \ ------^   A 
            
 (82) PC082: AGGAGAGUUUUAGUGUAGCUG
 ¦¤G = -63.60       
UG   UU   -   A      UAG  U    GAAGGAA     AAACA      .-U        .-UCCUUC| G 
  AUU  CAU AGG GAGUUU   UG AGCU       UAAGA     AGUAAA   GAUGCAUC        CA C
  UAA  GUA UCC CUCAAG   AC UCGA       AUUCU     UCAUUU   CUACGUAG        GU C
UA   CU   C   -      ---  -    AUGUUG-     -----      \ -        \ ------^ C 

(83) PC083: CGAAAGAAGAUAUAGAUGGUGUA
 ¦¤G = -22.60                
UUUUCUCCCAC           U        -      UU-|   AUG 
           GAAAGAAGAUA AGAUGGUG UAUUUU   UUUA   G
           CUUUUUUCUGU UUUGCUAC GUAAAA   AAAU   A
AAUUUUUAGAU           U        A      UUU^   AAU 
  
 (84) PC084: UAAGAAAGAACAAGUGUAGCAUA 
ÄG = -20.50 
            
AUUAUAUAU     -         GA   .-AGG    UU-    UACUU--|   C     AAUACUU 
         UUGUG UAUAUUUGU  UUU     UCUU   GUAA       UGGC UUUGU       U
         AAUAC AUGUGAACA  AAA     AGAA   CAUU       ACUG AAAUA       G
UCAAAAAAU     G         AG   \ ---    UGC    UUUUUUU^   U     ACUAUAA 
            
 (85)PC085: AGACUAUCCCUGAUUUGAGCAUA 
¦¤G = -37.30 
              
          CU       CC--    UUU-   -| UAA 
--AGAAUUUU  AGACUAU    CUGA    GAG CA   \
  UUUUAGAG  UCUGGUA    GAUU    UUC GU   U
\         UU       CGAA    UGAU   A^ CAG 
        
 (86)PC086: GCUGAGACUAAAACGGCUUGAUA 
¦¤G = -30.60 
                
AAC|      GAG      C           A     A 
   UUUUAUU   CAAGCU UUUUAGUCUCA CGUUG G
   AAAAUAA   GUUCGG AAAAUCAGAGU GUAAU A
UGA^      AUA      C           C     C 
       

(87) PC087: AGAGGAAAGGAUGAGAGAGAA
 ¦¤G = -52.00 
               
UACC    UU      -     U     A     C--     |     AAAA     UCUCU 
    CUUA  AGAGGA AAGGA GAGAG GAAAC   CCU--GAGUGA    GUUGU     A
    GAGU  UCUCUU UUUCU CUUUU UUUUG   GGA  CUCACU    CAACG     A
AUUA    UU      C     -     A     AUA   \ ^     ----     UGUGC 
  

(88) PC088: AGGGAUCUACUUUUGUAUUCAA
¦¤G = -54.10 
               
      UUU  C         C--  UU    UC-      A   -    ---   -|  UUU    AAAAACUCCAA 
--AUGU   UU AGGGAUCUA   UU  GUAU   AAAAUC AGU UUUG   GGU UAU   UAGC           \
  UGCA   AA UUUCUGGAU   AA  UAUG   UUUUGG UCA AGAC   CCA AUA   AUCG           U
\     UAU  -         UUC  UU    UUA      A   U    UCU   C^  UAU    AUAUAUUUAUA 
    
(89) PC089: AAGAGCAUUGGUGGCAAGACA
¦¤G = -51.40 
            
CUAU|    C         U    G   G  C        C      UAGAAAC 
    AGUUA UGUCUUGCC CCGA GCU UU ACGAAGAU UGCAUC       \
    UCAAU ACAGAACGG GGUU CGA AA UGCUUCUA ACGUAG       A
GCUU^    C         U    A   G  C        A      UAGAAAA 

 (90) PC090: UCUCAACCAUUGGAGAUGCUCUA
¦¤G = -70.40 
                 
CU   A--      UCA-          AUG    .-AA   C        CGU   U-     AAAAAAGA-     A    GA   .-ACAUUA|      AA 
  CCA   UGGGUC    ACCAUUGGAG   CUCU    CAA ACUUAUUU   ACG  AGCAU         GAGAG GAGA  GGU        AGGGCUC  G
  GGU   GCUCGG    UGGUAACUUU   GAGA    GUU UGAAUAAA   UGU  UUGUA         CUCUC CUCU  CCA        UUCUGGG  A
UU   ACA      UUGG          AA-    \ --   C        CC-   UU     AUCCUAGAG     -    G-   \ ------^      AG 
                     100  

 
    
(91) PC091: AUGGUUUAGAAGGACGGUCACUA
¦¤G = -18.30 
            
CUCGUAU         GAA-            GAUCCAAUUA-| U 
       UUUUGGUGA    UUUUCUAGACUA           GA G
       AGAAUCACU    GGAAGAUUUGGU           CU U
UUGUUAU         GGCA            AUUGAAUAGCA^ U 
   
£¨92£©PC092£ºAGAACGACACUCUUUGUGAAACA
ÄG = -72.30 
U      C   CA                         U        .-CACACAAAU|       AAAA 
 UACUC CUC  UUUCACAAAGAGUGUCGUUCU ACAUUUUU           UAAGAAAU    U
 AUGAG GAG  AAAGUGUUUCUCACAGCAAGA UGUGAAAA           AUUCUUUG    A
U      A   AC                         C        \ ---------^       GAAA 

£¨93£©PC093: AUGAGUACUUAAGAAGACCCA   
¦¤G = - 18.7
-      GGU--    -   -|  UAA     C  ACC 
 UUGUUC     GAUG AGU ACU   GAAGA CC   C
 AACGAG     UUAC UCA UGG   CUUCU GG   C
C      AGGUU    U   U^  CAA     A  AGA  


£¨94£©PC094: UAGAAAAACUUGAAUGGUGGAUA
¦¤G = -62.70 
AUA     A      U  UAA    -     A    .-CAU       .-C      -     AU--|   UAUUA   A  A       A           C-        A    CA      AAGAG   GUG----     GAAAAA 
   UCUAU UUAUCC CC   GUUC AGUUU UUUA     UUCUAUG   UAUUAU AAUAA    UAAG     AAU AA UAAAGGA GAAAGAGAAAA  AAAAAGAG AAAA  AGAAAA     AUU       UGAGA      A
   AGAUA GAUAGG GG   UAAG UCAAA AGAU     AAGAUAC   AUAAUA UUAUU    AUUC     UUA UU AUUUUCU CUUUCUUUUUU  UUUUUCUC UUUU  UCUUUU     UAA       AUUCU      A
AUG     G      U  ---    U     A    \ ---       \ -      U     AUAU^   UCAC-   C  A       -           AU        C    --      AUUA-   AAAAUUA     AGAUAA 

£¨95£©PC095: AGAAGAAGUAUAGGGUUUAGAUA
¦¤G = -25.30 
UGUAAAUA          UAU         G  .-AU       AGGAG----|         AAAAA 
        UAAGAAGAAG   AGGGUUUA AU    UAAAUAU         UUAAUUGUUU     \
        AUUUUUCUUU   UUCCAAAU UA    GUUUAUA         GAUUAAUAAA     A
ACCUUUUA          U--        G  \ --       AAAAUAGUA^         GUAUU

£¨96£©PC096: GAAUGGAAAAAACGACGAUGGUA
¦¤G = -59.60 
AAUUGCUA   C        A  AA          .-AUUU        .-U            .-UUUC      GC  A    CC         .-UUAGUUU|   U 
        UUU CUAUUGUU GU  UUUCCAUUCU      GUUUGUCU   UUCUU--CAUGA      CUUUAA  UU AUUU  UUUGUUGAU         CUGU U
        AAA GGUAGCAG CA  AAAGGUAAGA      CAGAUAGA   AAGAA  GUACU      GAAAUU  AA UAAA  AAACGACUA         GACA A
GAGAAAAA   U         -  AA          \ ----        \ -     \      \ ----      AA  A    --         \ -------^   A

£¨97£©PC097: UAAGAACUCUAAGAGCCAUUAGA
¦¤G = -29.70 
CUAAGA    UA        A       CA    AG C -|   A 
      GCUC  AGAACUCU AGAGC  UUAG  C C UAAG \
      CGAG  UCUUGAGA UUUCG  AAUC  G G AUUC G
UUUGUA    UA        A     AG    CU A A^   U

£¨98£©PC098: AACAGGAAUUGUAGGGAAGCAUA
ÄG = -43.30 
U   UU-    AA      --|        AG      UC     CC     CCAC  A      CUU    A    A    --   UU    U 
 UAU   UGUG  CAGGAA  UUGUAGGGA  CAUAAA  UGUUG  UUGUU    AU GAAUUA   GCAU CAAA UAUA  UUC  UACA U
 AUA   AUAU  GUUUUU  AACAUCCCU  GUGUUU  ACAAU  AACAA    UG UUUAGU   UGUA GUUU AUAU  AAG  AUGU U
U   UAU    AG      UC^        CU      --     UU     UUUA  A      U--    C    G    UA   U-    U

£¨99£©PC099: UUUGGAGCAAAAGUACAUUUUUA
ÄG = -49.70    
AGAGAA    A      CGA  CA          .-UAG     GA  U       -     .-UAAAAU|     AGA 
      UAUC GAGAAU   AC  UUGUUCUAAA     AGAAU  AG UGAAAAC UAUAC        UUAUUA   \
      AUAG UUUUUA   UG  AACGAGGUUU     UUUUG  UC ACUUUUG AUAUG        AAUAGU   A
AUUUAA    A      CA-  AA          \ ---        AC  -       U     \ ------^     ACG

£¨100£©PC100: AGGGUUCUAGAGCUAGAGUUCGG
¦¤G = -34.70 
GCUAAAAA          ----  G          AGGG   -   A---|    UU   CA    GG 
        CACUGGGCUU    UC GGAACUCUAA    AUC AUA    UCAGU  CAA  CUAG  \
        GUGGCUUGAG    AG UCUUGGGAUU    UAG UAU    AGUUA  GUU  GAUU  G
UCAGUAUG          AUCG  A            AUA-   C   AGAC^    GG    A-    UU

£¨101£©PC1016: CGAGAGACUUGACCGCGGUACA
¦¤G = -39.70 
-|                GA   GA      -   CAU      GG 
 GAUUUUUGGCCGAGA  CUU  CCGCG GUA   GUUUUU  C
 CUAAGAGCCGGUUUU  GAA  GGCGC CAU   CGAAAG  C
U^              AG   G-     U   ---      AG

£¨102£©PC102: AUAGGAGAAUUGAGGUAUAUCUA
¦¤G = -21.20     
UC    UUUC  A    AA      -  U  C   -   .-AUUUAAAAACAU|  A   A 
  UGAC    AU GGAG  UUGAGG UA AU UAA AGG              GGA AUG \
  ACUG    UA CCUC  AACUCU AU UA AUU UCC              CCU UAC U
UA    UU--  C    AA      G  C  U   C   \ ------------^  C   G

£¨103£©PC103: UUAAGACGUUCACUAAUAUUC
¦¤G = -50.60 
-                  CGU                  ---    GA-     A--|   C 
 UUCUUUCAUAUUAAGA   UCACUAAUAUUCAGU   GCGG   AGCGA   CGUU A
 AAGAAAGUAUAAUUCU   AGUGAUUGUAAGUCA   CGUC   UCGUU   GCAA C
G                   AUU                  CAU    ACA     GAA^   U

£¨104£©PC104: CUUCCGGAAACUUUGUGCUGA
¦¤G = -100.60GAGUA             CU A               C  C  .-GC     CAACAC                  --------     G    U   C 
               UCAGCACAAAGUU  C GGAGUUUUGCCACCA CA CG    GACCA      AAGCUAUGGUUAUGAGAU        UGAUC AUGG GAG U
               AGUCGUGUUUCAA  G CUUCGAGACGGUGGU GU GC    CUGGU      UUCGAUACCAAUACUCUA        ACUAG UACC CUU A
          AA---             AG C               C  C  \ --     C-----                  ACUUACUA     -    U   G

£¨105£©PC105: AUAAAACAAGAACGAGGGCAA
¦¤G = -166.80 
A   C     A                     G    A  C  CUC   G                C                     UAAC   U         AUU    -|            A                CU   UGAAUUUG  U 
 UCU GGAGU UAAAACAAGAACGAGGGCAAA AGCA UG AC   GCC GACAUAAUUCUACUCA ACUCUACUUUCAACUGCAAUC    UCU AGCUUAAAC   UAUU UUUUUUUGUUCUU UUCUUGUAAACUCUCU  AGG        AC U
 AGA CCUUA AUUUUGUUUUUGCUCUCGUUU UCGU AC UG   CGG UUGUGUUAAGAUGAGU UGAGAUGAAAGUUGACGUUAG    AGA UCGAGUUUG   AUAG AAAGAAACAAGAA AAGAAUAUUUGAGAGA  UCC        UG U
C   A     C                     A    C  C  AAA   A                A                     UUCA   U         AGU    A^            C                AC   UAAUAUUA  U

£¨106£©PC106:ACUGGAAUCUCCCCAUAGCUA
ÄG = -110.20 
-      .-AAU   U        C   U                       U    U      --        .-UC| U   AA 
 GAGCAU     CAC GGAAUCUC CCA AGCUAUCG--UAUGAAUUCUCCC GGGA AUAACA  AUUCUGGA    GC AGA  \
 CUCGUA     GUG CCUUAGAG GGU UCGAUAGC  AUACUUAAGAGGG CCCU UAUUGU  UAAGACCU    CG UCU  A
U      \ ---   U        U   U        \              C    U      UU        \ --^ U   AG

£¨107£©PC107: UGUAUGGAUUUUCAGAAGGCAUA
ÄG = -42.30 
CA          U  AUG       A--   -    UAAA    .-GU   -| AA 
  AUAGUUCG GU   GAUUUUC   GAA GGCA    AUGC    GGA GG  U
  UGUCAGGU CA   CUAAAAG   CUU CCGU    UGCG    CCU CC  A
GG        U  AA-       GAG   G    UC--    \ --   G^ GG

£¨108£©Pc108: GACUGGACUUGUGUUGAUUGGC
¦¤G = -51.30 
G  UC   -     UG--            .-CC|   GU 
 GC  CUG AGGUU    AACAUAGGUCCA    GGGG  G
 CG  GAC UCCGG    UUGUGUUCAGGU    CUCC  A
A  U-   U     UUAG            \ --^   AA

£¨109£©PC109£ºUGAAUCCAGGAGGUGGAAUGGUA
¦¤G = -35.40    
-     -      GAA   A  A-    -       AAUCA  G   G      ---| U 
 GCUGA AAAGAU   UCC GG  GGUG GAAUGGU     AG CUU AAAAAU   GG G
 CGGCU UUUUUG   AGG CC  UCGC CUUACCG     UU GAG UUUUUG   UC A
C     C      GA-   A  AG    U       GCUA-  G   G      UGU^ A

£¨110£©PC110£ºAGAGAAAAUUUGGUUACUGUUUA
¦¤G = -52.50 
UUCC      -        C  AG    C-     .-GAA      .-AA|   GCA        CAUCU   C    A   CCC 
    AAAAGA AGACAGUG AU  CAAA  UUCUC     AGUUCU    UAUA   AUCAAUAU     GGA AGUA AUU   U
    UUUUUU UUUGUCAU UG  GUUU  AAGAG     UCAAGA    AUAU   UAGUUAUA     CUU UUAU UAA   A
AAAU      A        -  --    AA     \ ---      \ --^   AA-        AUUAU   C    G   AAA

£¨111£©PC111£ºUCAAGAACAUUGAUAUCGUU
¦¤G = -24.00
AU---------        U      G           UAAU 
             AUGAUAUU GUGUUU UUGA--CCAAG    \
             UGCUAUAG UACAAG AACU  GGUUU    U
  UUCUAGAAAUU        U      -    \      UCAC

The red means the mature miRNAs locating in the 5' of the pre-miRNA sequences, and the blue means the mature miRNAs locating in the 3' of the pre-miRNA sequences.
