# Supplementary material for: Genome-wide identification of Thellungiella salsuginea microRNAs with putative roles in the salt stress response
Source: BMC Plant Biol. 2013 Nov 15;13:180. doi: 10.1186/1471-2229-13-180 (PMC4225614; doi:10.1186/1471-2229-13-180)
Supplement: Additional file 9: Table S9 — Stem-loop qRT-PCR primers used in the study of tsa-miRNA expression. [file 1471-2229-13-180-S9.doc]

**Table S9 Stem-loop qRT-PCR primers used in the study of tsa-miRNAs expression**

| **MiRNAs** | **Primer name** | **Sequence** |
| --- | --- | --- |
| tsa-miR156a | stem-loop RT primer | GTCGTATCCAGTGCAGGGTCCGAGGTATTCGCACTGGATACGACGTGCTC |
|  | forward primer | CGGCTGACAGAAGAGAG |
| tsa-miR157a | stem-loop RT primer | GTCGTATCCAGTGCAGGGTCCGAGGTATTCGCACTGGATACGACGTGCTC |
|  | forward primer | CGGCTTGACAGAAGATAGA |
| tsa-miR159a | stem-loop RT primer | GTCGTATCCAGTGCAGGGTCCGAGGTATTCGCACTGGATACGACGCTCTA |
|  | forward primer | CGGCTTTGGATTGAAGGGA |
| tsa-miR160a | stem-loop RT primer | GTCGTATCCAGTGCAGGGTCCGAGGTATTCGCACTGGATACGACTATGCA |
|  | forward primer | CGGCGCGTATGAGGAGCCA |
| tsa-miR166m | stem-loop RT primer | GTCGTATCCAGTGCAGGGTCCGAGGTATTCGCACTGGATACGACTTCCCT |
|  | forward primer | CGGCTCGGACCAGGCTTCA |
| tsa-miR167a | stem-loop RT primer | GTCGTATCCAGTGCAGGGTCCGAGGTATTCGCACTGGATACGACGATCTA |
|  | forward primer | CGGCTGAAGCTGCCAGCAT |
| tsa-miR168a | stem-loop RT primer | GTCGTATCCAGTGCAGGGTCCGAGGTATTCGCACTGGATACGACCGGGAA |
|  | forward primer | CGGCTCGCTTGGTGCAGGT |
| tsa-miR169a | stem-loop RT primer | GTCGTATCCAGTGCAGGGTCCGAGGTATTCGCACTGGATACGACTGCCGA |
|  | forward primer | CGGCCAGCCAAGGATGACT |
| tsa-miR169c | stem-loop RT primer | GTCGTATCCAGTGCAGGGTCCGAGGTATTCGCACTGGATACGACCGGCAA |
|  | forward primer | CGGCTGAGCCAAAGATGAC |
| tsa-miR390a | stem-loop RT primer | GTCGTATCCAGTGCAGGGTCCGAGGTATTCGCACTGGATACGACAGCGCC |
|  | forward primer | CGGCAAGCTCAGGAGGGAT |
| tsa-miR408 | stem-loop RT primer | GTCGTATCCAGTGCAGGGTCCGAGGTATTCGCACTGGATACGACGCCAGG |
|  | forward primer | CGGCATGCACTGCCTCTTC |
| tsa-miR894 | stem-loop RT primer | GTCGTATCCAGTGCAGGGTCCGAGGTATTCGCACTGGATACGACGGTGAA |
|  | forward primer | CGGCCGTTTCACGTCGGG |
| PC065 | stem-loop RT primer | GTCGTATCCAGTGCAGGGTCCGAGGTATTCGCACTGGATACGACATAAG |
|  | forward primer | CGGCAGTGACTTATAATACT |
| PC070 | stem-loop RT primer | GTCGTATCCAGTGCAGGGTCCGAGGTATTCGCACTGGATACGACTGCCT |
|  | forward primer | CGGCTGAAGGATCGAGGTCG |
| PC073 | stem-loop RT primer | GTCGTATCCAGTGCAGGGTCCGAGGTATTCGCACTGGATACGACTCATCT |
|  | forward primer | CGGCTGGAAAGTGAACAAA |
| Universal | reverse primer | GTGCAGGGTCCGAGGT |
| U6 | forward primer | GATAAAATTGGAACGATACAG |
|  | reverse primer | ATTTGGACCATTTCTCGATTT |
